# Prevalence and risk factors for painful diabetic peripheral neuropathy: a systematic review and meta-analysis

**DOI:** 10.3389/fneur.2025.1564867

**Published:** 2025-05-13

**Authors:** Peng Zhou, Jin song Zhou, Jin jie Li, Lang Qin, Wen feng Hu, Xiao yi Zhang, Jian xia Wang, Zhen Shi

**Affiliations:** ^1^The First Clinical Medical School, Hubei University of Chinese Medicine, Wuhan, China; ^2^Affiliated Hospital of Hubei University of Chinese Medicine, Wuhan, China; ^3^Hubei Provincial Hospital of Traditional Chinese Medicine, Wuhan, China; ^4^Hubei Provincial Institute of Traditional Chinese Medicine, Wuhan, China

**Keywords:** diabetes mellitus (DM), painful diabetic peripheral neuropathy (PDPN), risk factor, systematic review, meta-analysis

## Abstract

**Objective:**

This study aimed to explore the prevalence and risk factors for painful diabetic peripheral neuropathy through meta-analysis, and to provide countermeasures for early intervention and active prevention of diabetic peripheral neuropathic pain.

**Methods:**

We conducted a systematic search of PubMed, Embase, Web of Science, and Cochrane Library for articles published from the establishment of the database to November 2024. A total of 14 studies (nine cross-sectional studies and five cohort studies) qualified for meta-analysis. We used a random-effects meta-analysis and assessed heterogeneity and publication bias.

**Results:**

A total of 14 studies (nine cross-sectional, five cohort) were included. The prevalence of PDPN was estimated at 33.9% (95% CI [19.4%−48.5%]). Significant risk factors included female gender (OR = 1.29, *P* = 0.004), glycosylated hemoglobin levels (WMD = 0.14, *P* < 0.001), nephropathy (OR = 1.41, *P* < 0.001), retinopathy (OR = 1.32, *P* = 0.040), cardiovascular disease (OR = 1.46, *P* < 0.001), arterial hypertension (OR = 1.25, *P* = 0.047), triglycerides levels (WMD = −0.10, *P* < 0.001), low density lipoprotein levels (WMD = −0.20, *1*. 0.001), smoking (OR = 0.90, *P* = 0.001), drinking alcohol (OR = 0.87, *P* = 0.001), glomerular filtration rate levels (WMD = −7.11, *P* < 0.001), obesity (OR = 2.00, *P* = 0.001). The multivariate analysis results showed that female gender (OR = 1.42, *P* = 0.032), age of onset (OR = 1.19, *P* < 0.001), duration of diabetes (OR = 1.29, *P* < 0.001), and retinopathy (OR = 1.90, *P* = 0.032) were indicated as risk factors for painful diabetic peripheral neuropathy.

**Conclusion:**

This meta-analysis suggests that female gender, glycosylated hemoglobin levels, nephropathy, retinopathy, cardiovascular disease, arterial hypertension, smoking, drinking alcohol, glomerular filtration rate levels, obesity, age of onset and duration of diabetes are indicated as risk factors for painful diabetic peripheral neuropathy in diabetic patients.

**Systematic review registration:**

https://www.crd.york.ac.uk/PROSPERO/, identifier CRD42025629060.

## 1 Background

Diabetes mellitus (DM) is one of the chronic diseases that afflicts all age groups around the world, the associated neuropathy is its most costly and disabling complication (1, 2), affects up to 50% or more of patients (3, 4). And the prevalence of PDPN is increasing year by year and getting younger and younger (1, 5). PDPN, also known as diabetic neuropathic pain. For diabetic patients, PDPN occurs in about 50% of diabetic patients (6, 7). Despite its common nature, PDPN is underdiagnosed and undertreated and is often the main reason for medical visits. The prevalence PDPN determined in a community-based study is about 26% (8). However, the incidence of PDPN is far higher than this. PDPN is usually characterized by distal lower limb pain (9, 10). It was characterized by burning pain, tearing pain and stinging pain (10). In addition to presenting as unbearable pain, it may be accompanied by problems such as poor sleep quality (11)and mood disorders (12, 13). At present, the treatment of PDPN is challenging (14–16). Although Alabdali et al. (17) showed that anticonvulsant, antidepressant, and opioid drugs reduced pain in patients with PDPN to some extent, a series of adverse effects brought by pain symptoms significantly reduced the quality of life of patients. At present, there are more and more studies on diabetic peripheral neuropathy, and more and more evidence shows that it is related to advanced glycation terminal pathway (2), however, the pathophysiological, biochemical, molecular and pharmacological mechanisms of PDPN are not fully understood. Therefore, it is of great positive significance to explore the related influencing factors of PDPN. This study explores the prevalence and risk factors for PDPN through Meta-analysis and systematic review and resolves the controversy about the risk factors of PDPN, in order to improve the quality of life and prognosis of patients. It is an important update for clinicians to provide treatment and prevention of PDPN.

## 2 Materials and methods

This systematic review is conducted according to the preferred reporting items of the Protocol for Systematic Reviews and Meta-analyses (PRISMA-P) guidelines. The review will be conducted according to PRISMA criteria (18). The registration number is CRD42025629060.

### 2.1 Search strategy

PubMed, Embase, Web of Science, and Cochrane Library were searched by computer for articles on influencing factors of diabetic patients with peripheral neuropathic pain. The search time limit was from the establishment of the database to November 2024. A combination of subject headings and free words was used to search, and references of included studies were also searched to supplement the acquisition of relevant information (see Supplementary Tables 1–4 for specific retrieval strategies).

### 2.2 Literature inclusion and exclusion criteria

Inclusion criteria: (1) patients with confirmed diagnosis of diabetes type 1 or type 2 (19); (2) the influencing factors of diabetic peripheral neuropathy pain in diabetic patients in the study; (3) study types were case-control study, cohort study or cross-sectional study; (4) outcome indicators were whether diabetes patients were complicated with PDPN and related measurement data.

Exclusion criteria: (1) non-English literature; (2) review literature, conference papers, case reports, etc. (3) repeated publications; (4) articles without full text or with low quality evaluation.

### 2.3 Literature data extraction

Two researchers independently screened the literature and extracted the data according to the inclusion and exclusion criteria of the literature. If there were differences of opinion, the third researcher participated in the consultation and discussion to decide whether to be included. Original study authors were contacted by mail if they did not report important information needed for the study. The data extracted included the first author, publication year, study type, country, sample size, number of PDPN cases, PDPN diagnostic criteria, regression model and influencing factors.

### 2.4 Literature quality evaluation

Two researchers independently evaluated the quality of the included studies using the Newcastle-Ottawa scale (NOS) (20). The NOS scale assesses the quality of literature from three aspects: population selection, comparability between groups and exposure assessment. The total score of NOS is 9 points, and it is generally considered that the score of NOS 0–3 is low quality, 4–6 is moderate quality, and 7–9 is high quality. If two researchers disagreed during the evaluation process, the decision would be discussed, or a third party would decide.

### 2.5 Statistical methods

Stata15.0 software was used to perform a meta-analysis of the extracted data. The continuous variables were expressed as weighted mean difference (WMD) and 95% confidence interval (CI), and the dichotomous variables were expressed as odds ratio (OR). The positive and negative signs of WMD indicate the direction, and the value indicates the actual clinical effect. An OR > 1 indicates an increased risk. *P* value and I^2^ were used to judge the heterogeneity among the studies. If *P* ≥ 0.10 and I^2^ < 50%, there was no significant heterogeneity among the studies, and the fixed effect model was used for analysis. If *P* < 0.10 and I^2^ ≥ 50%, it indicated that there was significant heterogeneity among the studies. Subgroup analysis was performed on the year of publication, country and region, study type, literature quality score, and pain questionnaire adopted by PDPN diagnosis to explore the suspected sources of heterogeneity (see Supplementary Figures 1–7, Supplementary Table 1). A random effect model was used for data analysis. For I_2_ > 50%, the sensitivity analysis was performed by the test method of elimination one by one (see Supplementary Figures 8–12), and Egger's test was used to analyze the publication bias (see Supplementary Tables 5, 6). The significance level was set at 0.05.

## 3 Results

### 3.1 Results of literature search

**Figure 1:**
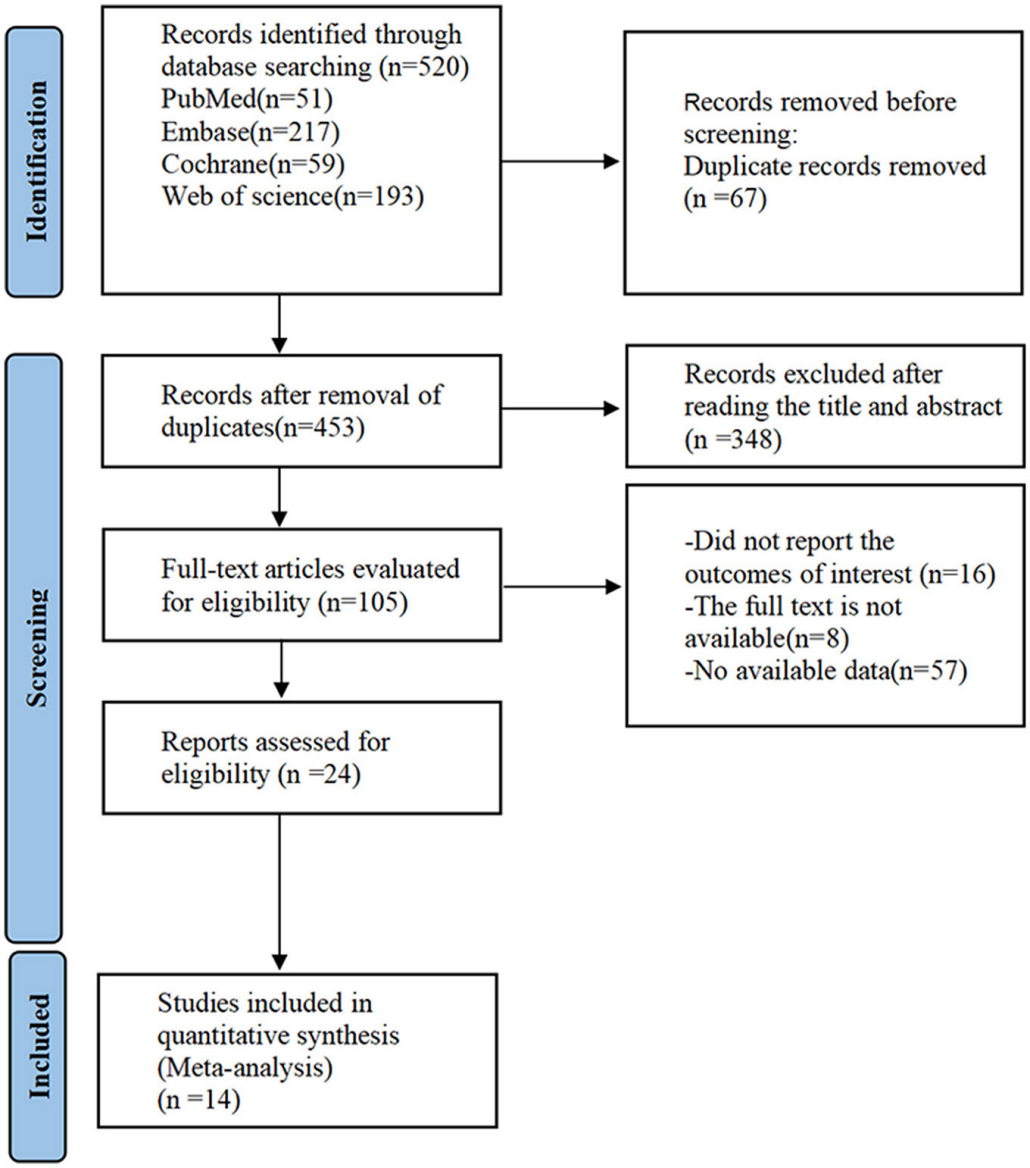
Flow chart of the literature search.

By searching PubMed, Embase, Cochrane Library, and Web of Science databases, 520 documents were initially obtained, 453 documents were retained after removing duplicate documents, 348 articles were preliminarily screened by reading the titles and abstracts, and 14 documents were included after reading the full text (see Figure 1).

### 3.2 Basic features of the included literature

A total of 14 studies (21–34) were included, of which 5 were cohort studies (23, 24, 26, 28, 32) and 9 were cross-sectional studies (21, 22, 25, 27, 29–31, 33, 34). There were 16,829 patients with PDPN and 17,281 patients with painless diabetic peripheral neuropathy. The age of the study ranged from 0 to 96 years (see Supplementary Tables 7, 8). The included 14 articles were evaluated by NOS quality evaluation, and two of them scored 6 points, indicating that the study quality was moderate. The remaining scores were 7–8, and the overall quality of the studies included was high (see Supplementary Table 9 for specific quality evaluations).

### 3.3 Prevalence of painful diabetic peripheral neuropathy

**Figure 2:**
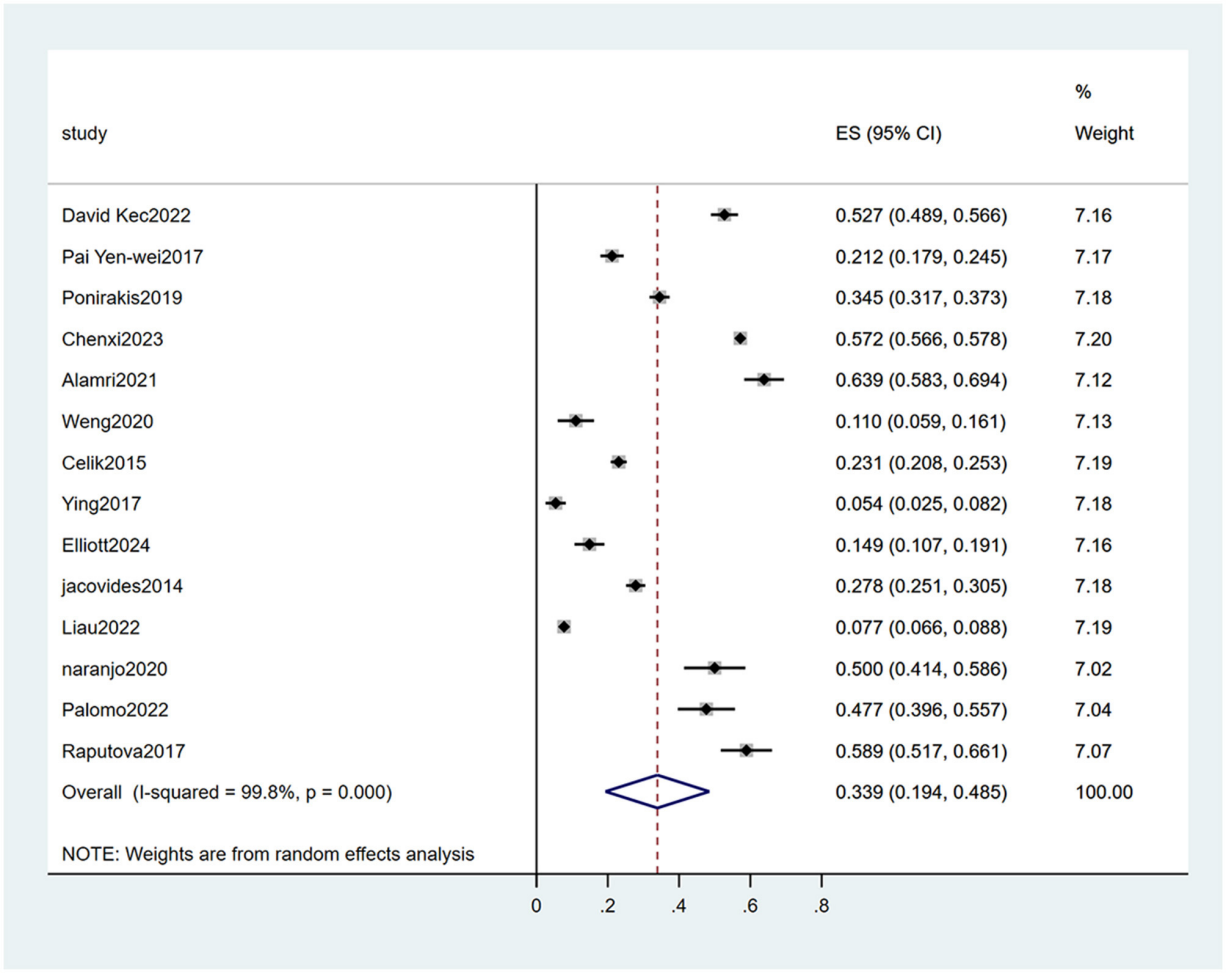
Forest plot of the meta-analysis of the prevalence of PDPN.

**Figure 3:**
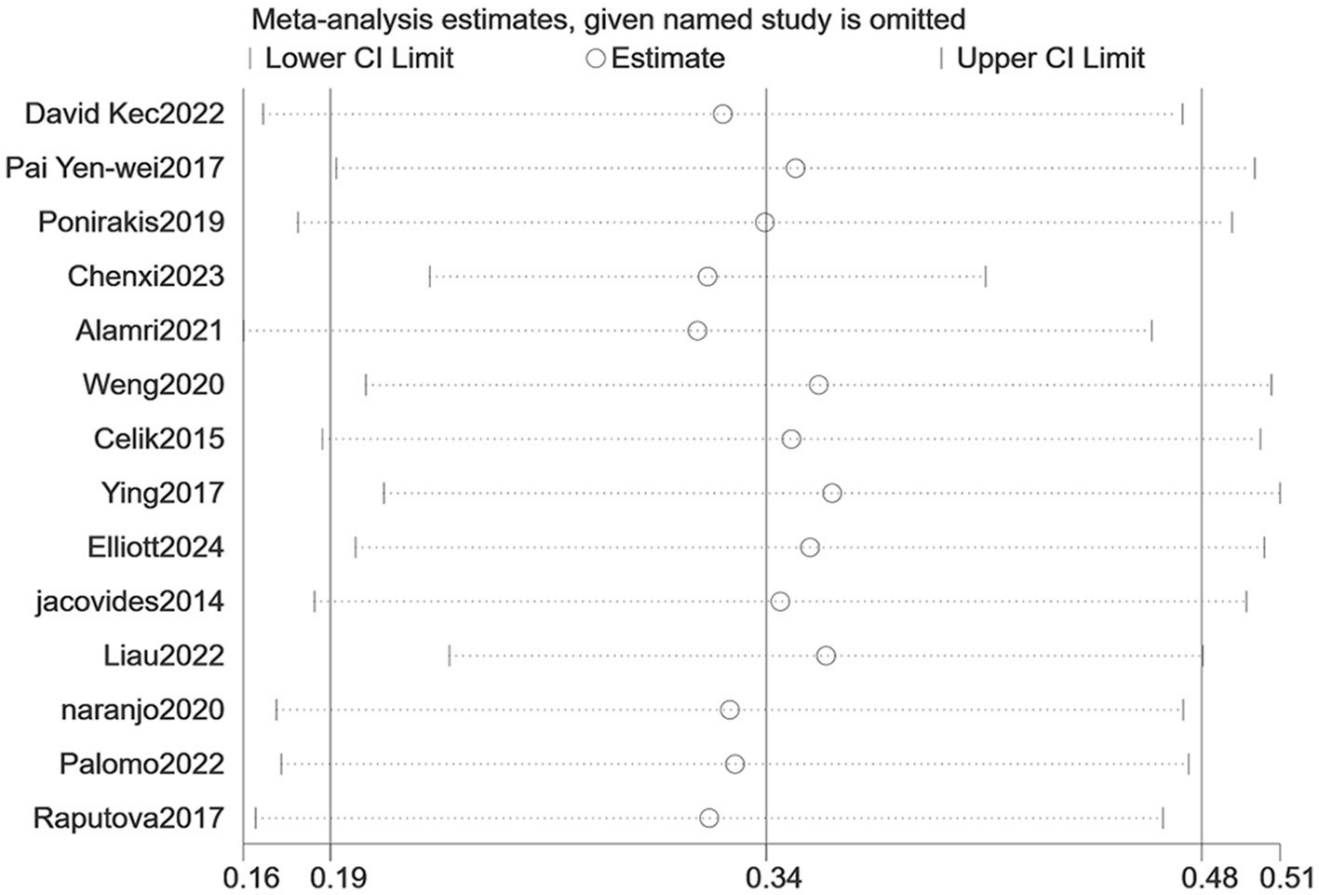
Sensitivity analysis of the prevalence of PDPN.

**Figure 4:**
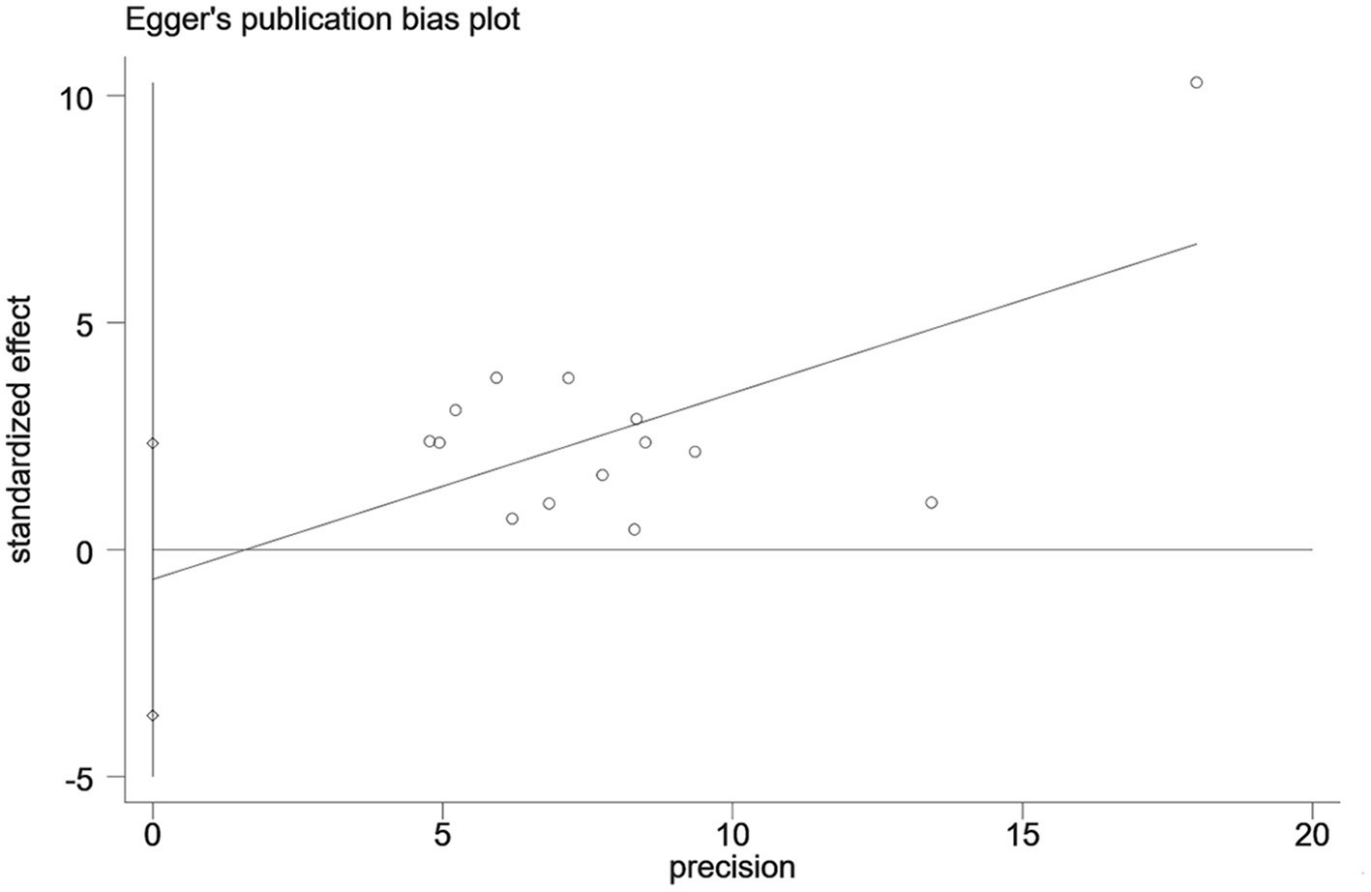
Egger test of the prevalence of PDPN.

The heterogeneity test (I_2_ = 99.8%, *P* < 0.001) was conducted based on the random-effects model. It was found that the prevalence of PDPN was estimated at 33.9% (95% CI [19.4%-48.5%]). The analysis results indicated that sensitivity was low, and the analysis results were stable. The Egger test was performed on the index to evaluate publication bias (*P* = 0.642), and the prevalence may not have been subject to publication bias (see Figures 2–4).

### 3.4 Results of single factors meta-analysis

#### 3.4.1 Female gender

**Figure 5:**
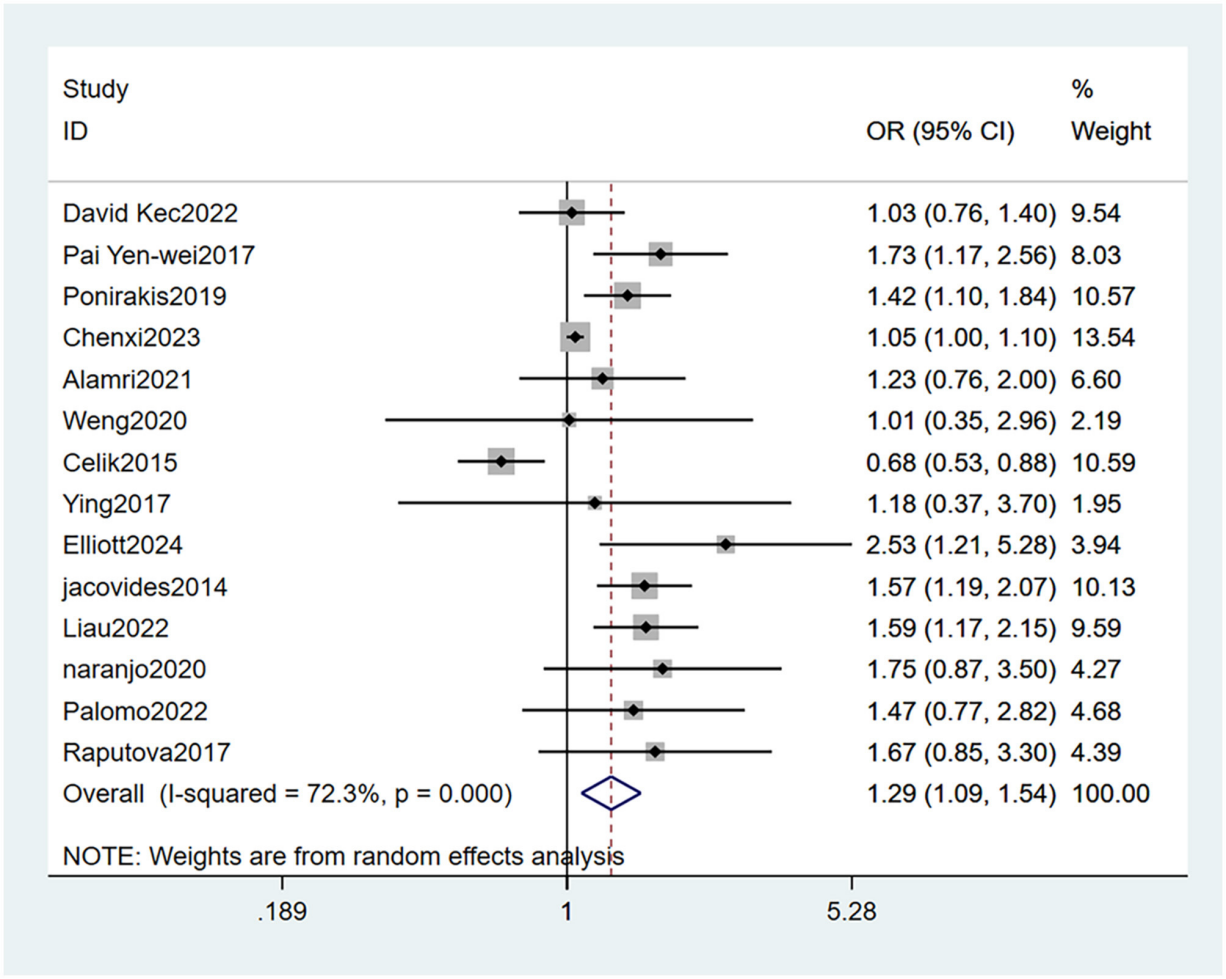
Forest plot of the meta-analysis of female gender.

Fourteen studies (21–34) mentioned female as a risk factor for PDPN, and the heterogeneity test (I_2_ = 72.3%, *P* < 0.001) was performed based on the random-effects model. It was found that female was considered a statistically significant risk factor for PDPN (OR = 1.29, 95% CI [1.09,1.54], *P* = 0.004) (see Figure 5, Supplementary Table 5).

#### 3.4.2 Glycosylated hemoglobin levels

**Figure 6:**
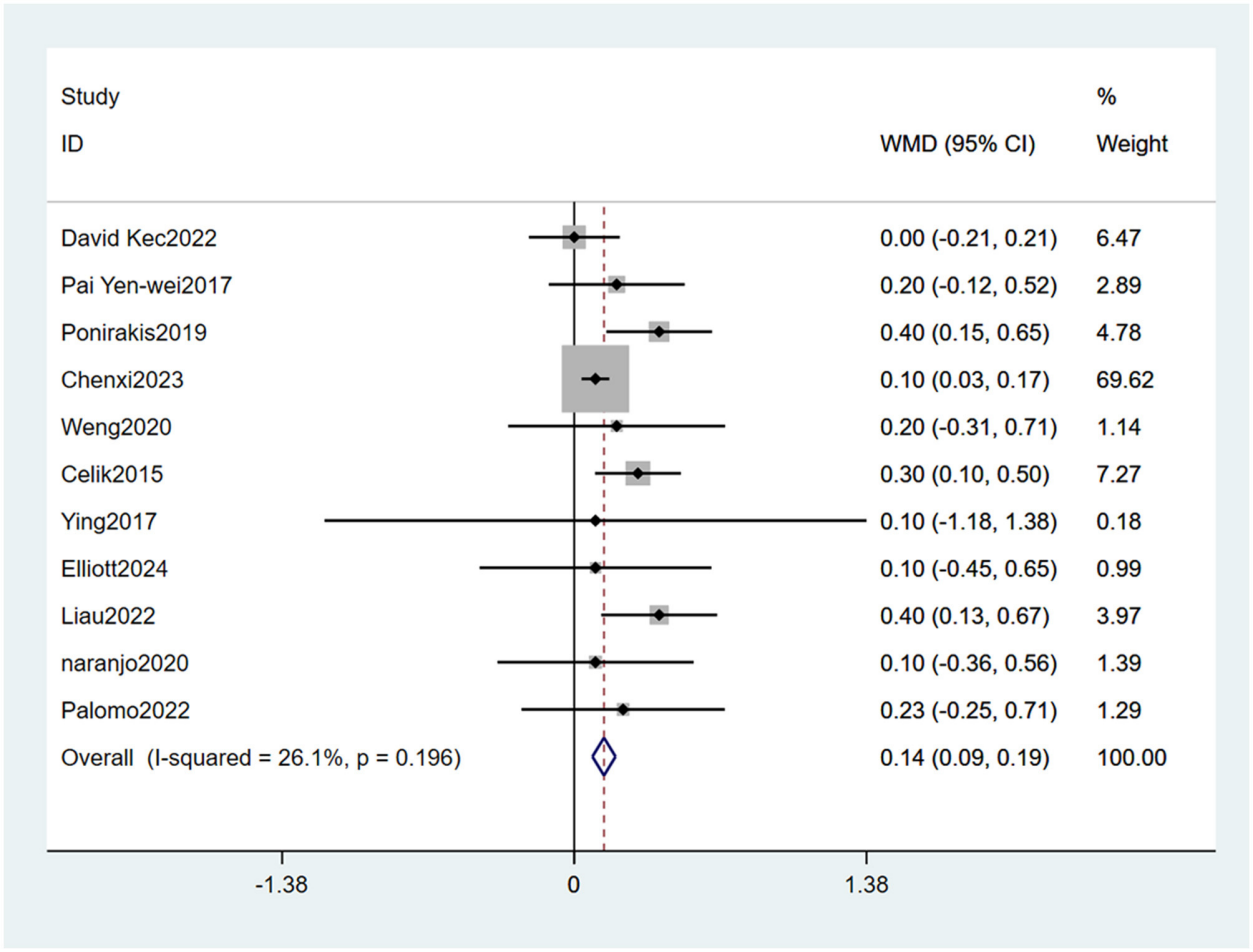
Forest plot of the meta-analysis of glycosylated hemoglobin levels.

Eleven studies (22–24, 26–32, 34) mentioned glycosylated hemoglobin levels as a risk factor, and the heterogeneity test (I_2_ = 26.1%, *P* = 0.196) was performed based on the fixed-effects model. It was found that glycosylated hemoglobin levels was considered a statistically significant risk factor for PDPN (OR = 0.14, 95% CI [0.09, 0.19], *P* < 0.001) (see Figure 6, Supplementary Table 5).

#### 3.4.3 Concomitant nephropathy

**Figure 7:**
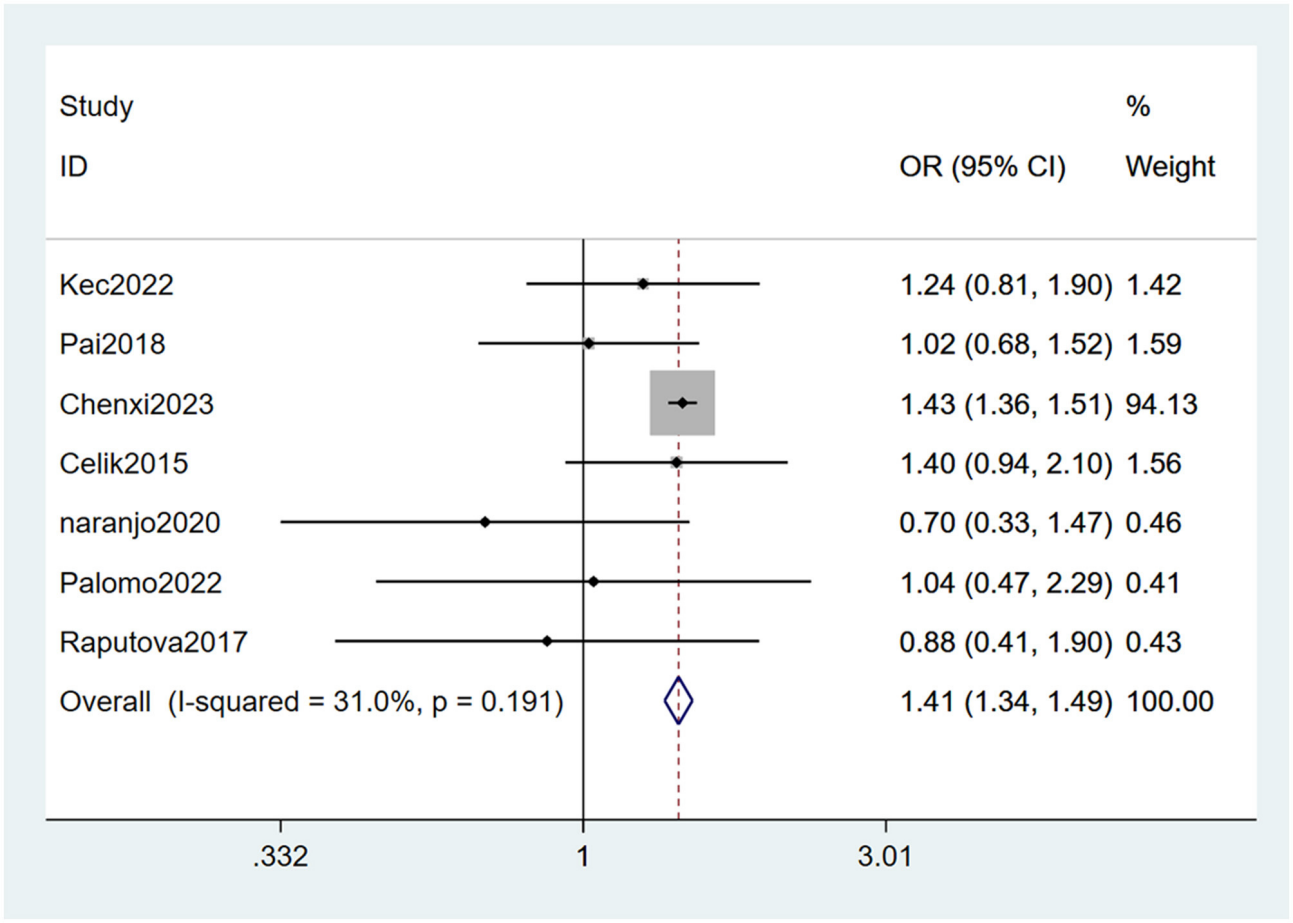
Forest plot of the meta-analysis of concomitant nephropathy.

Nephropathy (22, 26, 27, 29–31, 33) was mentioned as a risk factor for PDPN, and the heterogeneity test (I_2_ = 31.0%, *P* = 0.191) was performed based on the fixed-effects model. It was found that nephropathy was considered a statistically significant risk factor for PDPN (OR = 1.41, 95% CI [1.34, 1.49], *P* < 0.001) (see Figure 7, Supplementary Table 5).

#### 3.4.4 Associated retinopathy

**Figure 8:**
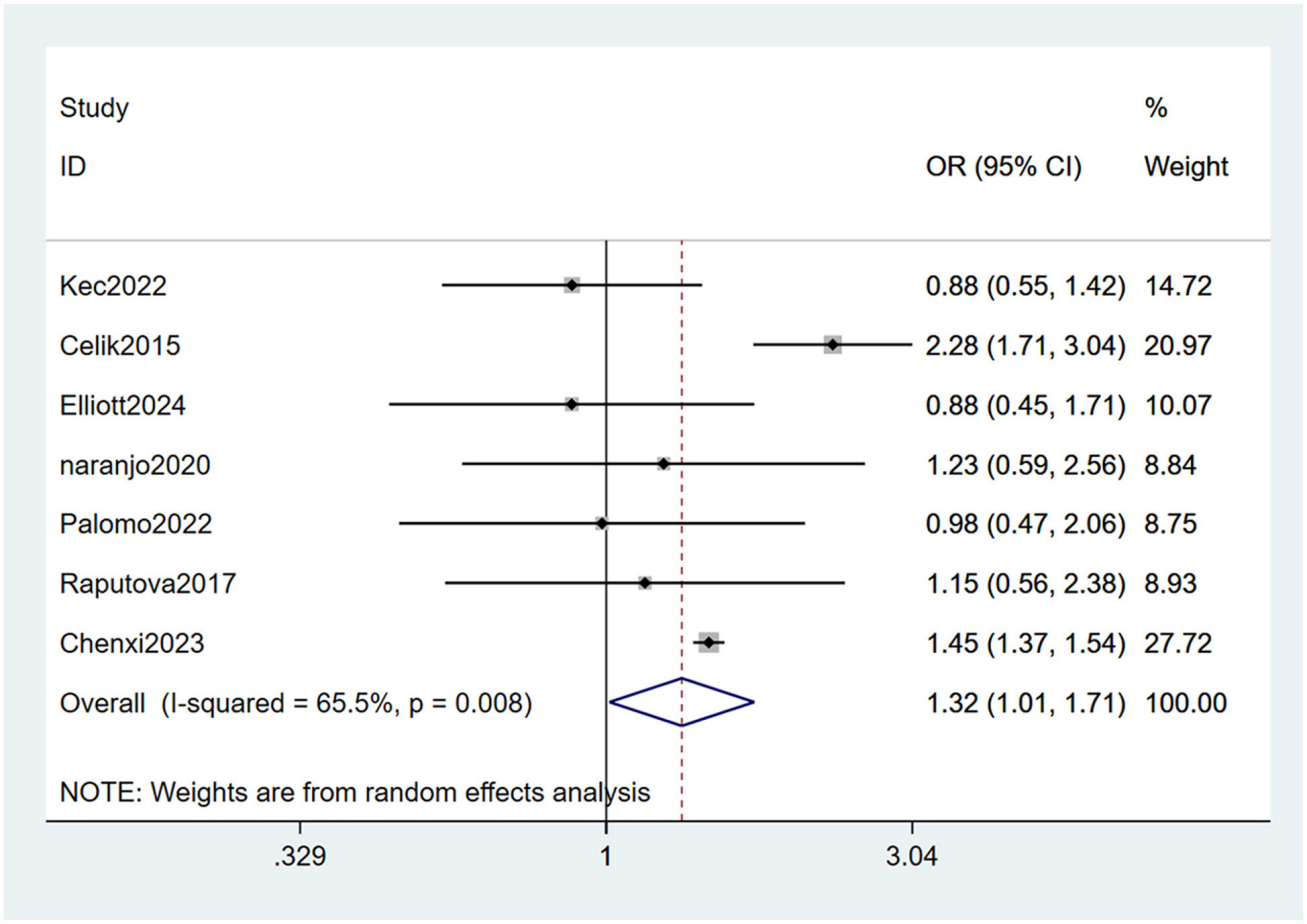
Forest plot of the meta-analysis of associated retinopathy.

Seven studies (22, 23, 26, 27, 29, 31, 33) mentioned retinopathy as a risk factor for PDPN, and the heterogeneity test (I_2_ = 65.5%, *P* = 0.008) was performed based on the random-effects model. It was found that retinopathy was considered a statistically significant risk factor for PDPN (OR = 1.32, 95% CI [1.01, 1.71], *P* = 0.040) (see Figure 8, Supplementary Table 5).

#### 3.4.5 Concomitant cardiovascular disease

**Figure 9:**
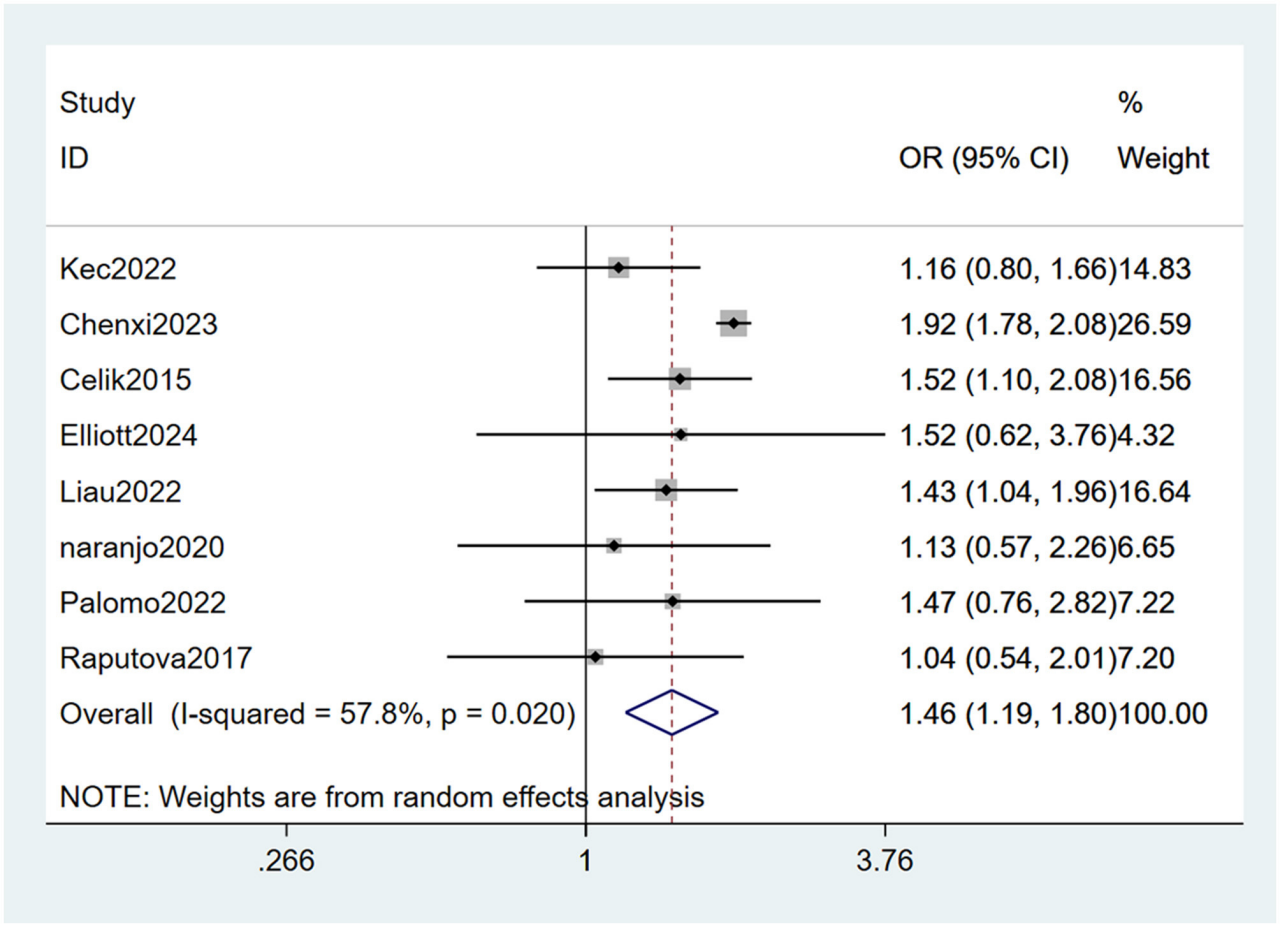
Forest plot of the meta-analysis of concomitant cardiovascular disease.

Cardiovascular disease (22, 23, 26–29, 31, 33) was mentioned as a risk factor in eight studies, and the heterogeneity test (I_2_ = 57.8%, *P* = 0.020) was performed based on the random-effects model. It was found that cardiovascular disease was considered a statistically significant risk factor for PDPN (OR = 1.46, 95% CI [1.19, 1.80], *P* < 0.001) (see Figure 9, Supplementary Table 5).

#### 3.4.6 Arterial hypertension

**Figure 10:**
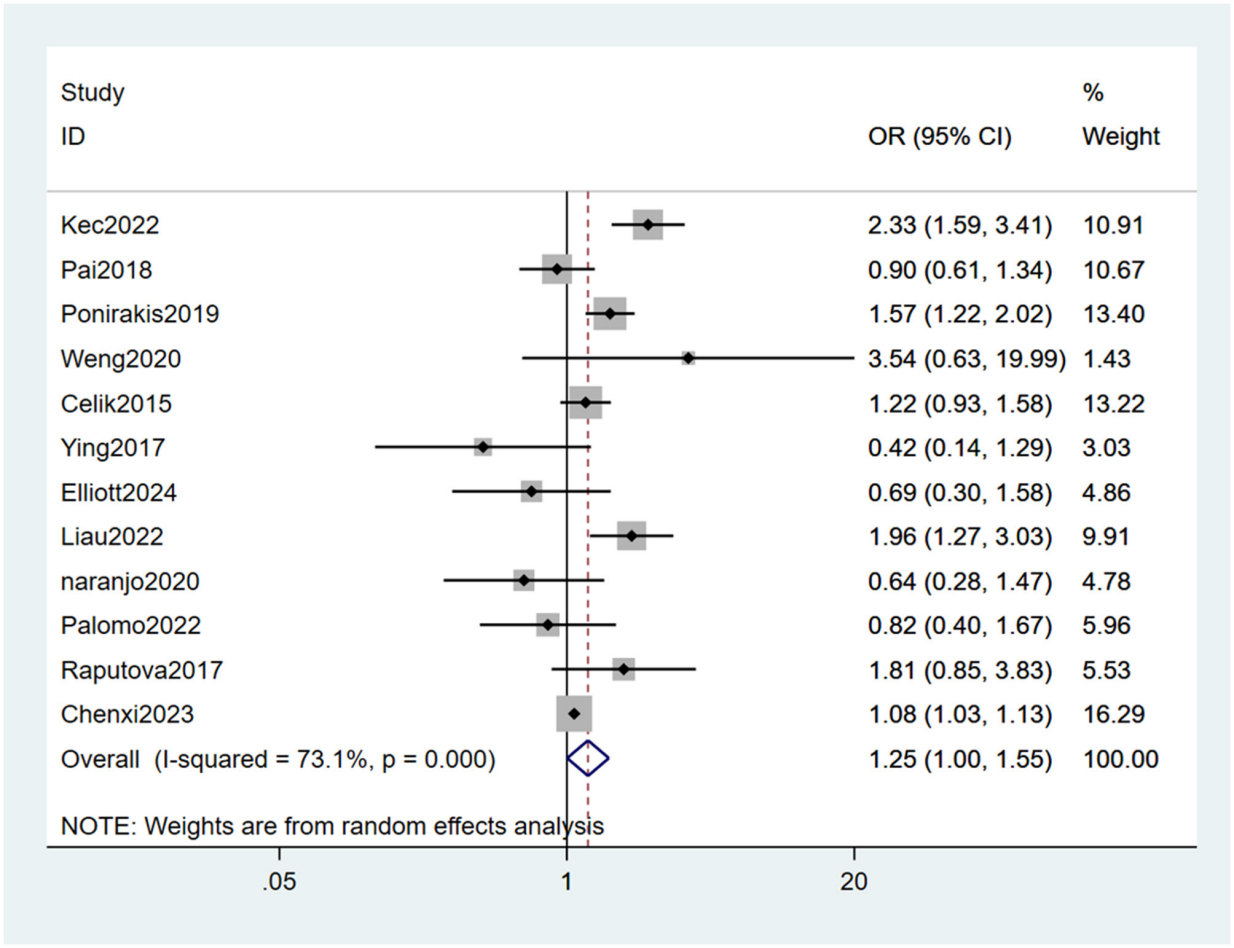
Forest plot of the meta-analysis of arterial hypertension.

Twelve studies (22–24, 26–34) mentioned arterial hypertension as a risk factor, and the heterogeneity test (I_2_ = 73.1%, *P* = 0.000) was performed based on the random-effects model. It was found that arterial hypertension was considered a statistically significant risk factor for PDPN (OR = 1.25, 95% CI [1.00, 1.55], *P* = 0.047) (see Figure 10, Supplementary Table 5).

#### 3.4.7 Triglycerides levels

**Figure 11:**
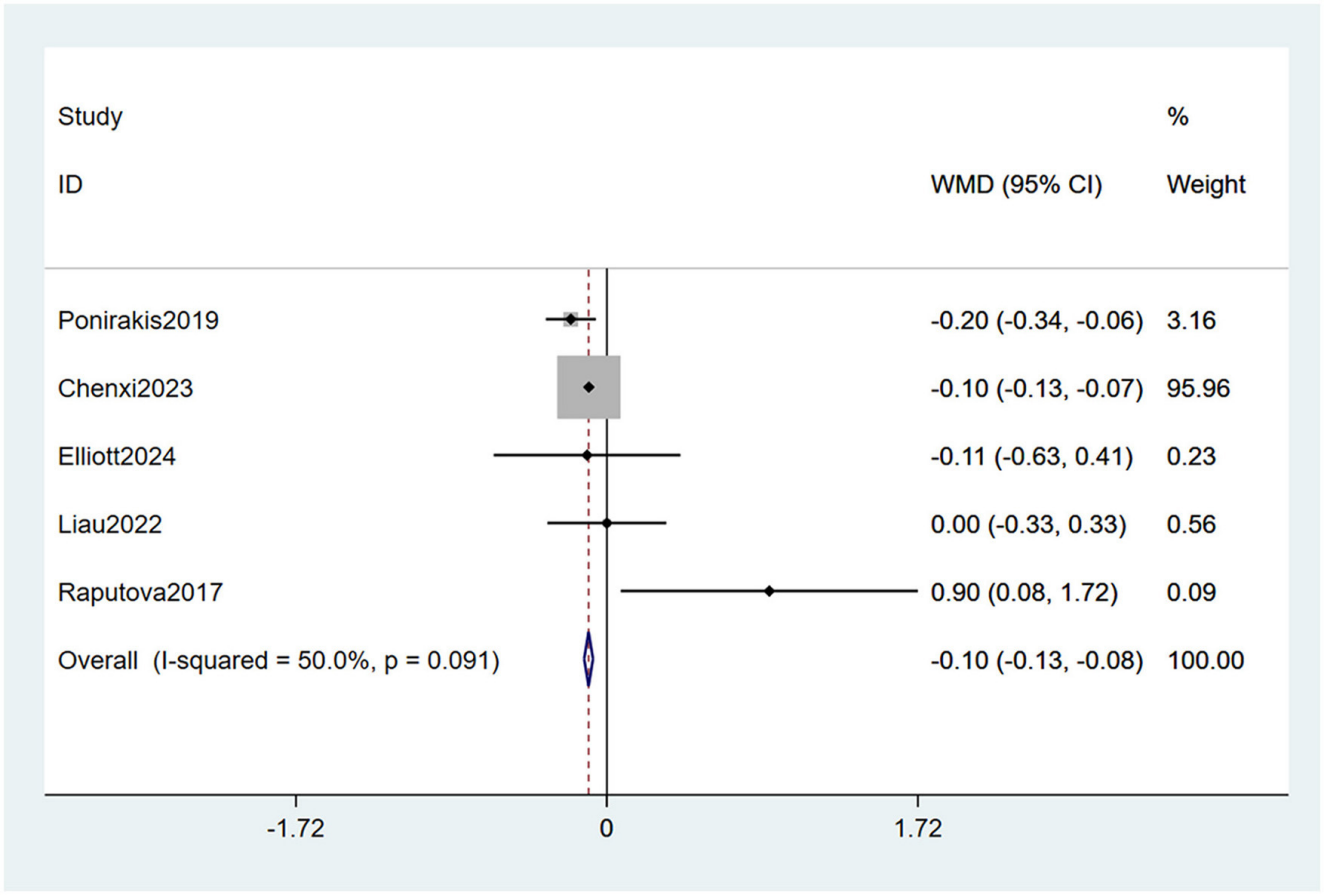
Forest plot of the meta-analysis of triglycerides levels.

Five studies (23, 27, 28, 32, 33) mentioned triglycerides levels as a risk factor, and the test (I2 = 50.0%, *P* = 0.091) indicates moderate heterogeneity. It was found that triglycerides levels were considered a statistically significant risk factor for PDPN (WMD = −0.10, 95% CI [−0.13, −0.08], *P* < 0.001) (see Figure 11, Supplementary Table 5).

#### 3.4.8 Low density lipoprotein levels

**Figure 12:**
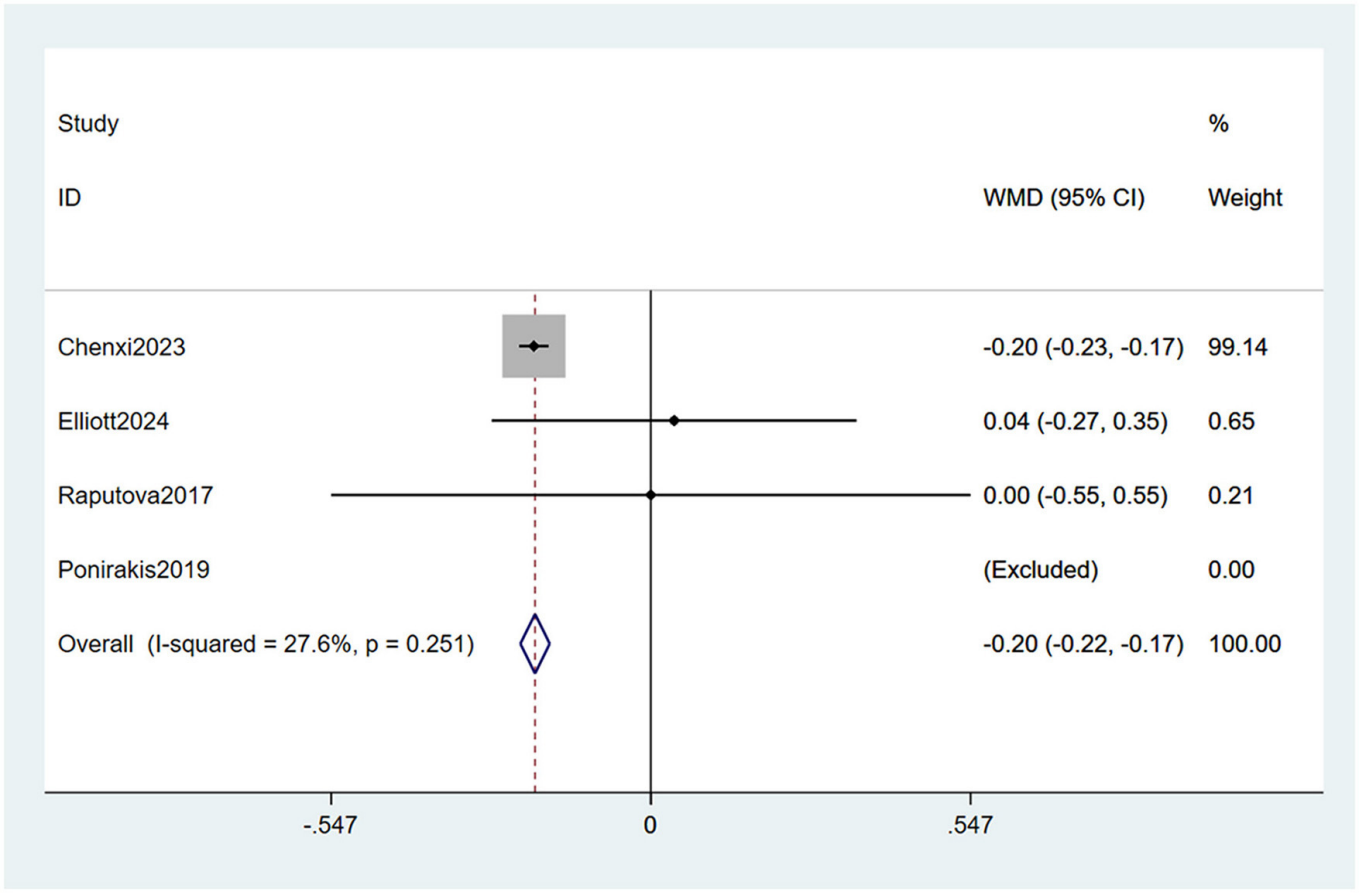
Forest plot of the meta-analysis of low density lipoprotein levels.

Four studies (23, 27, 32, 33) mentioned low density lipoprotein levels as a risk factor, and the heterogeneity test (I_2_ = 27.6%, *P* = 0.251) was performed based on the fixed-effects model. It was found that low density lipoprotein levels were considered a statistically significant risk factor for PDPN (WMD = −0.20, 95% CI [−0.22, −0.17], *P* < 0.001) (See Figure 12, Supplementary Table 5).

#### 3.4.9 Smoking

**Figure 13:**
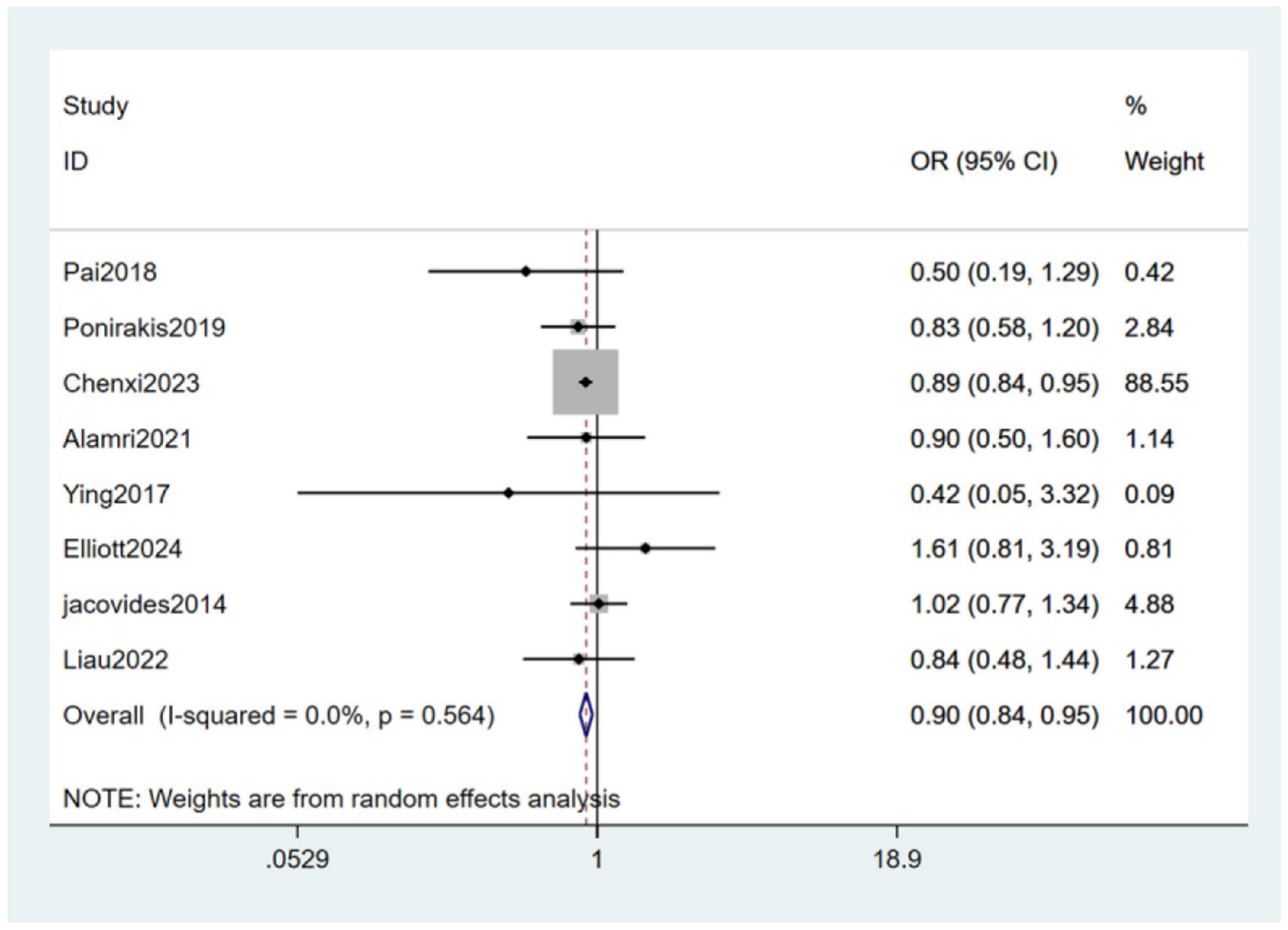
Forest plot of the meta-analysis of smoking.

Smoking (21, 23–25, 27, 28, 30, 32) was mentioned as a risk factor in eight studies, and the heterogeneity test (I_2_ = 0.0%, *P* = 0.564) was performed based on the fixed-effects model. It was found that smoking was considered a statistically significant risk factor for PDPN (OR = 0.90, 95% CI [0.84, 0.95], *P* = 0.001) (see Figure 13, Supplementary Table 5).

#### 3.4.10 Drinking alcohol

**Figure 14:**
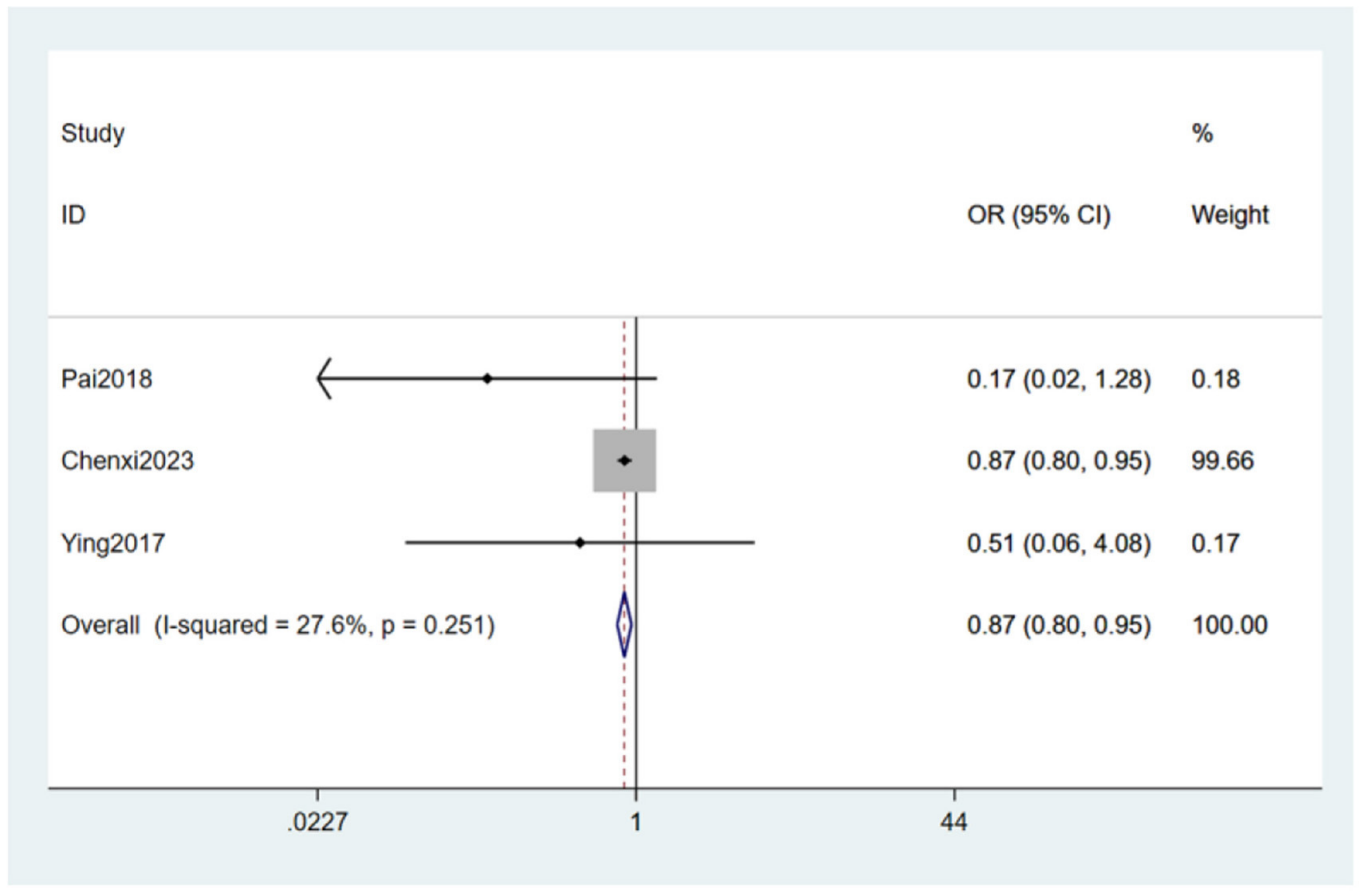
Forest plot of the meta-analysis of drinking alcohol.

Drinking alcohol (24, 27, 30) was mentioned as a risk factor in three studies, and the heterogeneity test (I_2_ = 27.6%, *P* = 0.251) was performed based on the fixed-effects model. It was found that drinking alcohol was considered a statistically significant risk factor for PDPN (OR = 0.87, 95% CI [0.80,0.95], *P* = 0.001) (see Figure 14, Supplementary Table 5).

#### 3.4.11 Glomerular filtration rate levels

**Figure 15:**
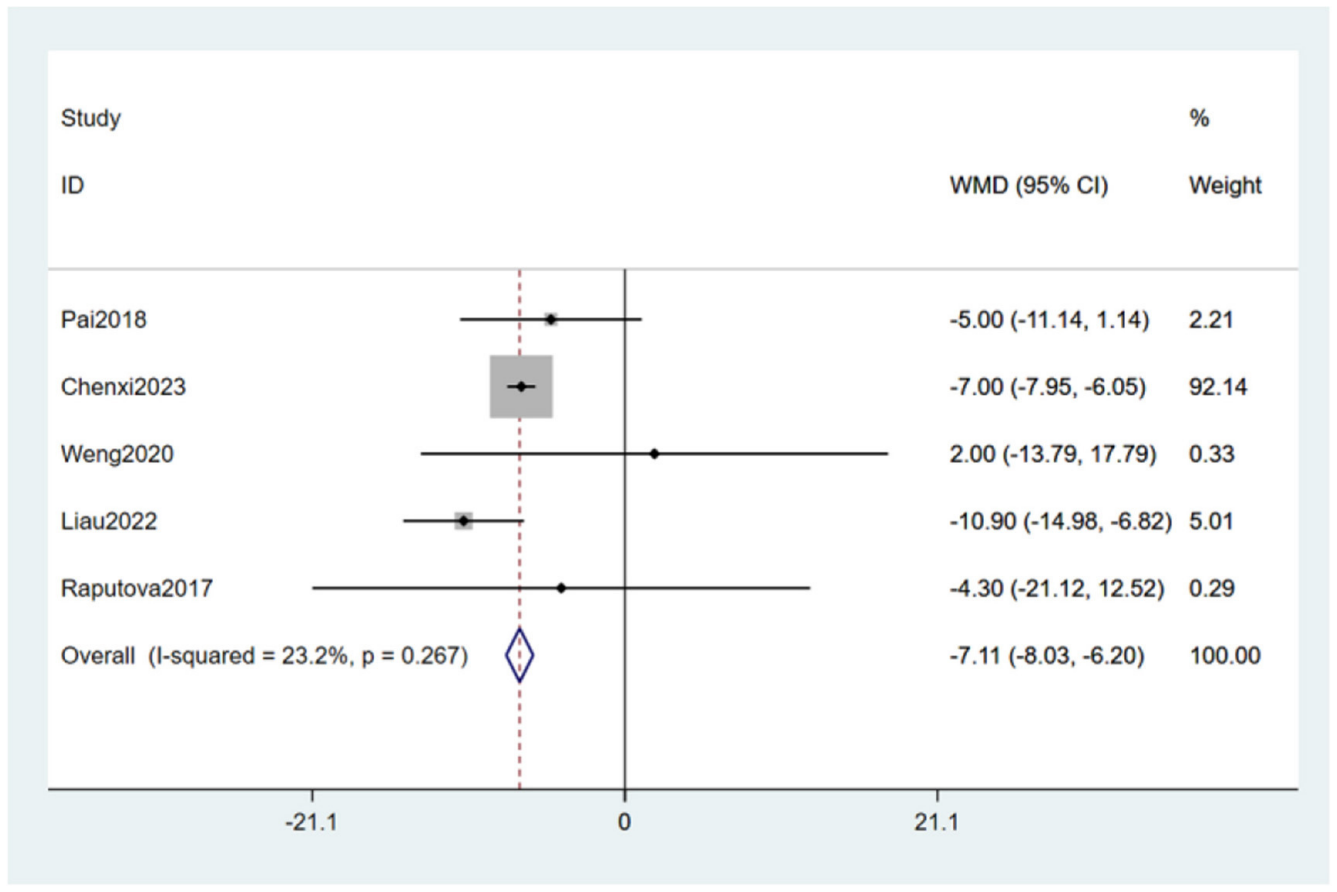
Forest plot of the meta-analysis of glomerular filtration rate levels.

Five studies (27, 28, 30, 33, 34) mentioned glomerular filtration rate levels as a risk factor, and the heterogeneity test (I_2_ = 23.2%, *P* = 0.267) was performed based on the fixed-effects model. It was found that glomerular filtration rate levels was considered a statistically significant risk factor for PDPN (WMD = −7.11, 95% CI [−8.03,-6.20], *P* < 0.001) (see Figure 15, Supplementary Table 5).

#### 3.4.12 Obesity

**Figure 16:**
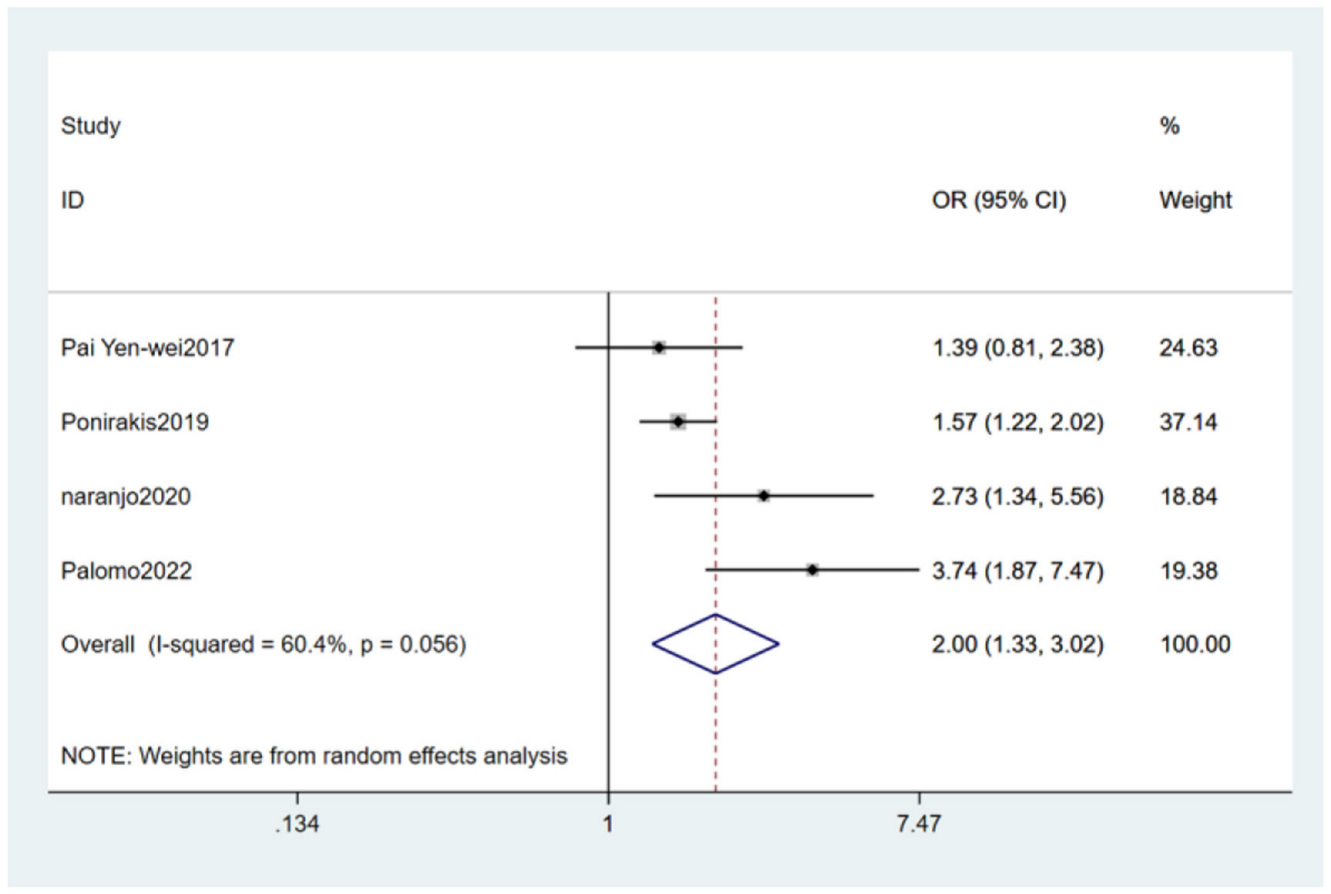
Forest plot of the meta-analysis of obesity.

Five studies (27, 29–32) mentioned obesity as a risk factor, and the heterogeneity test (I_2_ = 60.4%, *P* = 0.056) was performed based on the random-effects model. It was found that obesity was considered a statistically significant risk factor for PDPN (OR = 2.00, 95% CI [1.33, 3.02], *P* = 0.001) (see Figure 16, Supplementary Table 5).

#### 3.4.13 Other meta-analysis results

Differences in the correlations between BMI, dyslipidemia levels, cholesterol levels, high density lipoprotein levels, retirement, work regularly, oral hypoglycemic agents, lipid-lowering agents, insulin therapy, sports and exercise, diabetic foot ulcers and serum creatinine levels were not statistically significant (see Supplementary Table 5).

### 3.5 Results of multiple factors meta-analysis

The results of the multifactorial analysis mentioned in the research study were analyzed and combined. It was found that female gender (OR = 1.42, 95% CI [1.03, 1.97], *P* = 0.032), age of onset (OR = 1.19, 95% CI [1.11,1.27], *P* < 0.001), duration of diabetes (OR = 1.29, 95% CI [1.15, 1.45], *P* < 0.001) and retinopathy (OR = 1.90, 95% CI [1.06, 3.40], *P* = 0.032) were considered a statistically significant risk factor for PDPN (see Supplementary Table 6).

## 4 Publication bias

The egger test was used to evaluate the publication bias of each risk factor, and the *P* values of most indicators were >0.05, suggesting that almost all risk factors did not have publication bias (see Supplementary Tables 5, 6).

## 5 Discussion

Although many previous reports have used review and meta-analysis to report the prevalence of diabetic peripheral neuropathy, this study is the first to use meta-analysis to investigate the risk factors for PDPN, a subtype of diabetic peripheral neuropathy and one of the common chronic complications of diabetes. The prevalence of PDPN can be reduced by the risk factors described above, such as a lower probability of developing PDPN in persons without diabetic retinopathy than in those with retinopathy. PDPN is characterized by persistent pain in the lower extremity from bottom to top, distal to proximal, presenting with a range of neuropathic symptoms that include burning, deep pain, needling, and electric shock-like pain (11, 35). In addition to the underlying factors such as cultural, environmental and psychosocial factors, the pathogenesis of pain also includes peripheral structure, molecular biomarkers and the central nervous system (36), heredity (37) and gender (38). However, more attention is needed to clarify the pathogenesis of PDPN.

This study found that female is a risk factor for PDPN through univariate and multivariate analysis, indicating that female diabetic patients are more likely to have peripheral nerve pain than male diabetic patients, which is consistent with the results of previous studies (39, 40). The reason for this may be related to the emotional instability of female patients or may be affected by estrogen and thus have a low tolerance to pain. Huang et al. (41) also reported that about two thirds of female diabetic patients are susceptible to peripheral neuropathy. Recent studies have also highlighted female sex as a potential risk factor for PDPN (32, 38, 42), our study just adds to the evidence.

The present study also found that advanced age is a risk factor for PDPN, which is consistent with the results of Sang et al. (30) in a multi-center, large-sample cross-sectional study in Korea. The longer the duration of diabetes, the more likely it is to develop PDPN, this finding has been confirmed by other studies (32, 43, 44). The possible reason may be that the continuous high concentration of blood glucose leads to nerve damage (45), it is consistent with our study that PDPN is more likely to occur with higher Hba1c concentration. Therefore, it is necessary to strengthen the focus of attention and education for diabetic patients to prevent the occurrence of PDPN. It is a risk factor for PDPN in patients with arterial hypertension, which is consistent with the results of previous studies (40, 46, 47), the likely reason is that the cumulative effect of hypertension damages the myelin sheath of the nerve (48), PDPN is then induced. In addition, diabetic complications such as nephropathy, retinopathy, cardiovascular disease, hyperlipidemia levels, obesity and other comorbidities are not independent of each other, but mutually affect the occurrence of PDPN (39, 40, 49). Studies have shown that (50), diabetic nephropathy and retinopathy are independent risk factors for PDPN, which was again confirmed in our study. Studies have shown that (51, 52), smoking and alcohol consumption were associated with the occurrence of PDPN, again consistent with the conclusions obtained in our study. This study also found that oral hypoglycemic drugs, lipid-lowering drugs and insulin therapy were protective factors, suggesting that the occurrence of PDPN may not be related to clinical medication. Regular exercise was reported as a protective factor (53, 54), this is consistent with the conclusion of this study, which may be related to the fact that exercise can promote blood glucose metabolism and slow down nerve conduction. Although many risk factors for PDPN have been identified, there are still unknown risk factors for PDPN. Therefore, during clinical treatment, the patient's condition should be comprehensively considered, all risk factors leading to PDPN should be screened and prevented in advance as far as possible, and the early prevention and treatment of PDPN should be actively carried out to reduce the risk of PDPN and improve the prognosis of patients.

This study still has the following limitations: firstly, the number of articles included is small, and the types of articles are cross-sectional studies and cohort studies, which may have selection bias. Prospective studies may be needed in the future to avoid publication bias. Secondly, although no source of heterogeneity was identified by subgroup analysis, perhaps we can identify potential sources of heterogeneity in future studies. Thirdly, although the egger test did not show significant bias, publication bias does exist, studies with negative results may not have been published, leading us to overestimate the risk. Prospective studies and uniform diagnostic methods for PDPN may be needed in the future to avoid publication bias. Finally, because the original studies did not control confounders consistently (for example, no universal adjustment for socioeconomic status), the results of this meta-analysis may be affected by residual confounding.

## 6 Conclusion

Based on the available evidence, female gender, glycosylated hemoglobin levels, nephropathy, retinopathy, cardiovascular disease, arterial hypertension, triglycerides levels, low density lipoprotein levels, smoking, drinking alcohol, glomerular filtration rate levels, obesity, age of onset and duration of diabetes are identified as risk factors for PDPN, and clinical practitioners can combine these indicators for early detection, diagnosis, and intervention in such kind of cases, thereby improving the quality of life of affected individuals.

## Data Availability

The datasets presented in this study can be found in online repositories. The names of the repository/repositories and accession number(s) can be found in the article/Supplementary material.
