# Supplementary material for: Prevalence and risk factors for painful diabetic peripheral neuropathy: a systematic review and meta-analysis
Source: Front Neurol. 2025 May 13;16:1564867. doi: 10.3389/fneur.2025.1564867 (PMC12108811; doi:10.3389/fneur.2025.1564867)
Supplement: Supplementary file 1 [file Supplementary_file_1.docx]

**Table S1 ： PubMed search strategy**

| Search  number | Query | Results |
| --- | --- | --- |
| 1 | "Risk Factors"[Mesh] | 998,909 |
| 2 | ((((((((((((((((((Risk Factors[Title/Abstract]) OR (Factor, Risk[Title/Abstract])) OR (Risk Factor[Title/Abstract])) OR (Population at Risk[Title/Abstract])) OR (Populations at Risk[Title/Abstract])) OR (Risk Scores[Title/Abstract])) OR (Risk Score[Title/Abstract])) OR (Score, Risk[Title/Abstract])) OR (Risk Factor Scores[Title/Abstract])) OR (Risk Factor Score[Title/Abstract])) OR (Score, Risk Factor[Title/Abstract])) OR (Health Correlates[Title/Abstract])) OR (Correlates, Health[Title/Abstract])) OR (Social Risk Factors[Title/Abstract])) OR (Factor, Social Risk[Title/Abstract])) OR (Factors, Social Risk[Title/Abstract])) OR (Risk Factor, Social[Title/Abstract])) OR (Risk Factors, Social[Title/Abstract])) OR (Social Risk Factor[Title/Abstract]) | 888,309 |
| 3 | #1 OR #2 | 2,878,695 |
| 4 | "Diabetes Mellitus"[Mesh] | 537,621 |
| 5 | ((((((((((((((((((((((((((((Diabetes Mellitus, Type 2[Title/Abstract]) OR (Diabetes Mellitus, Adult-Onset[Title/Abstract])) OR (Adult-Onset Diabetes Mellitus[Title/Abstract])) OR (Diabetes Mellitus, Adult Onset[Title/Abstract])) OR (Diabetes Mellitus, Ketosis-Resistant[Title/Abstract])) OR (Diabetes Mellitus, Ketosis Resistant[Title/Abstract])) OR (Ketosis-Resistant Diabetes Mellitus[Title/Abstract])) OR (Diabetes Mellitus, Non Insulin Dependent[Title/Abstract])) OR (Diabetes Mellitus, Non-Insulin-Dependent[Title/Abstract])) OR (Non-Insulin-Dependent Diabetes Mellitus[Title/Abstract])) OR (Diabetes Mellitus, Stable[Title/Abstract])) OR (Stable Diabetes Mellitus[Title/Abstract])) OR (Diabetes Mellitus, Type II[Title/Abstract])) OR (Diabetes Mellitus, Noninsulin Dependent[Title/Abstract])) OR (Diabetes Mellitus, Maturity-Onset[Title/Abstract])) OR (Maturity-Onset Diabetes Mellitus[Title/Abstract])) OR (Maturity Onset Diabetes Mellitus[Title/Abstract])) OR (Diabetes Mellitus, Slow-Onset[Title/Abstract])) OR (Diabetes Mellitus, Slow Onset[Title/Abstract])) OR (Slow-Onset Diabetes Mellitus[Title/Abstract])) OR (Type 2 Diabetes Mellitus[Title/Abstract])) OR (Noninsulin-Dependent Diabetes Mellitus[Title/Abstract])) OR (Noninsulin Dependent Diabetes Mellitus[Title/Abstract])) OR (Maturity-Onset Diabetes[Title/Abstract])) OR (Diabetes, Maturity-Onset[Title/Abstract])) OR (Maturity Onset Diabetes[Title/Abstract])) OR (Type 2 Diabetes[Title/Abstract])) OR (Diabetes, Type 2[Title/Abstract])) OR (Diabetes Mellitus, Noninsulin-Dependent[Title/Abstract]) | 198,519 |
| 6 | #4 OR #5 | 1219,159 |
| 7 | "Neuralgia"[Mesh] | 26103 |
| 8 | (((((((((((((((((((((((((((((((((((((((((((((((Neuralgia[Title/Abstract]) OR (Neuralgias[Title/Abstract])) OR (Neurodynia[Title/Abstract])) OR (Neurodynias[Title/Abstract])) OR (Neuropathic Pain[Title/Abstract])) OR (Neuropathic Pains[Title/Abstract])) OR (Pain, Neuropathic[Title/Abstract])) OR (Pains, Neuropathic[Title/Abstract])) OR (Nerve Pain[Title/Abstract])) OR (Nerve Pains[Title/Abstract])) OR (Pain, Nerve[Title/Abstract])) OR (Pains, Nerve[Title/Abstract])) OR (Paroxysmal Nerve Pain[Title/Abstract])) OR (Nerve Pain, Paroxysmal[Title/Abstract])) OR (Nerve Pains, Paroxysmal[Title/Abstract])) OR (Pain, Paroxysmal Nerve[Title/Abstract])) OR (Pains, Paroxysmal Nerve[Title/Abstract])) OR (Paroxysmal Nerve Pains[Title/Abstract])) OR (Neuralgia, Atypical[Title/Abstract])) OR (Atypical Neuralgia[Title/Abstract])) OR (Atypical Neuralgias[Title/Abstract])) OR (Neuralgias, Atypical[Title/Abstract])) OR (Neuralgia, Iliohypogastric Nerve[Title/Abstract])) OR (Iliohypogastric Nerve Neuralgia[Title/Abstract])) OR (Iliohypogastric Nerve Neuralgias[Title/Abstract])) OR (Nerve Neuralgia, Iliohypogastric[Title/Abstract])) OR (Nerve Neuralgias, Iliohypogastric[Title/Abstract])) OR (Neuralgias, Iliohypogastric Nerve[Title/Abstract])) OR (Neuralgia, Ilioinguinal[Title/Abstract])) OR (Ilioinguinal Neuralgia[Title/Abstract])) OR (Ilioinguinal Neuralgias[Title/Abstract])) OR (Neuralgias, Ilioinguinal[Title/Abstract])) OR (Neuralgia, Perineal[Title/Abstract])) OR (Neuralgias, Perineal[Title/Abstract])) OR (Perineal Neuralgia[Title/Abstract])) OR (Perineal Neuralgias[Title/Abstract])) OR (Neuralgia, Stump[Title/Abstract])) OR (Neuralgias, Stump[Title/Abstract])) OR (Stump Neuralgia[Title/Abstract])) OR (Stump Neuralgias[Title/Abstract])) OR (Neuralgia, Supraorbital[Title/Abstract])) OR (Neuralgias, Supraorbital[Title/Abstract])) OR (Supraorbital Neuralgia[Title/Abstract])) OR (Supraorbital Neuralgias[Title/Abstract])) OR (Neuralgia, Vidian[Title/Abstract])) OR (Neuralgias, Vidian[Title/Abstract])) OR (Vidian Neuralgia[Title/Abstract])) OR (Vidian Neuralgias[Title/Abstract]) | 97052 |
| 9 | #7 OR #8 | 126,123 |
| 10 | #3 AND #6 AND #9 | 51 |

**Table S2 ： Embase search strategy**

| No. | Query | Results |
| --- | --- | --- |
| #103 | #31 AND #81 AND #102 | 217 |
| #102 | #82 OR #83 OR #84 OR #85 OR #86 OR #87 OR #88 OR #89 OR #90 OR #91 OR #92 OR #93 OR #94 OR #95 OR #96 OR #97 OR #98 OR #99 OR #100 OR #101 | 1702177 |
| #101 | 'social risk factor':ab,ti | 131 |
| #100 | 'risk factors, social':ab,ti | 157 |
| #99 | 'risk factor, social':ab,ti | 8 |
| #98 | 'factors, social risk':ab,ti | 10 |
| #97 | 'factor, social risk':ab,ti | 0 |
| #96 | 'social risk factors':ab,ti | 1500 |
| #95 | 'correlates, health':ab,ti | 12 |
| #94 | 'health correlates':ab,ti | 661 |
| #93 | 'score, risk factor':ab,ti | 17 |
| #92 | 'risk factor score':ab,ti | 303 |
| #91 | 'risk factor scores':ab,ti | 153 |
| #90 | 'score, risk':ab,ti | 1387 |
| #89 | 'risk score':ab,ti | 52756 |
| #88 | 'risk scores':ab,ti | 25074 |
| #87 | 'populations at risk':ab,ti | 3843 |
| #86 | 'population at risk':ab,ti | 6164 |
| #85 | 'risk factor':ab,ti | 425454 |
| #84 | 'factor, risk':ab,ti | 362 |
| #83 | 'risk factor':ab,ti | 425454 |
| #82 | 'risk factor'/exp | 1477162 |
| #81 | #32 OR #33 OR #34 OR #35 OR #36 OR #37 OR #38 OR #39 OR #40 OR #41 OR #42 OR #43 OR #44 OR #45 OR #46 OR #47 OR #48 OR #49 OR #50 OR #51 OR #52 OR #53 OR #54 OR #55 OR #56 OR #57 OR #58 OR #59 OR #60 OR #61 OR #62 OR #63 OR #64 OR #65 OR #66 OR #67 OR #68 OR #69 OR #70 OR #71 OR #72 OR #73 OR #74 OR #75 OR #76 OR #77 OR #78 OR #79 OR #80 | 154988 |
| #80 | 'vidian neuralgias':ab,ti | 0 |
| #79 | 'vidian neuralgia':ab,ti | 1 |
| #78 | 'neuralgias, vidian':ab,ti | 0 |
| #77 | 'neuralgia, vidian':ab,ti | 1 |
| #76 | 'supraorbital neuralgias':ab,ti | 1 |
| #75 | 'supraorbital neuralgia':ab,ti | 70 |
| #74 | 'neuralgias, supraorbital':ab,ti | 0 |
| #73 | 'neuralgia, supraorbital':ab,ti | 3 |
| #72 | 'stump neuralgias':ab,ti | 0 |
| #71 | 'stump neuralgia':ab,ti | 1 |
| #70 | 'neuralgias, stump':ab,ti | 0 |
| #69 | 'neuralgia, stump':ab,ti | 0 |
| #68 | 'perineal neuralgias':ab,ti | 3 |
| #67 | 'perineal neuralgia':ab,ti | 24 |
| #66 | 'neuralgias, perineal':ab,ti | 0 |
| #65 | 'neuralgia, perineal':ab,ti | 0 |
| #64 | 'neuralgias, ilioinguinal':ab,ti | 0 |
| #63 | 'ilioinguinal neuralgias':ab,ti | 6 |
| #62 | 'ilioinguinal neuralgia':ab,ti | 45 |
| #61 | 'neuralgia, ilioinguinal':ab,ti | 1 |
| #60 | 'neuralgias, iliohypogastric nerve':ab,ti | 0 |
| #59 | 'nerve neuralgias, iliohypogastric':ab,ti | 0 |
| #58 | 'nerve neuralgia, iliohypogastric':ab,ti | 0 |
| #57 | 'iliohypogastric nerve neuralgias':ab,ti | 0 |
| #56 | 'iliohypogastric nerve neuralgia':ab,ti | 0 |
| #55 | 'neuralgia, iliohypogastric nerve':ab,ti | 0 |
| #54 | 'neuralgias, atypical':ab,ti | 2 |
| #53 | 'atypical neuralgias':ab,ti | 9 |
| #52 | 'atypical neuralgia':ab,ti | 29 |
| #51 | 'neuralgia, atypical':ab,ti | 26 |
| #50 | 'paroxysmal nerve pains':ab,ti | 0 |
| #49 | 'pains, paroxysmal nerve':ab,ti | 0 |
| #48 | 'pain, paroxysmal nerve':ab,ti | 0 |
| #47 | 'nerve pains, paroxysmal':ab,ti | 0 |
| #46 | 'nerve pain, paroxysmal':ab,ti | 0 |
| #45 | 'paroxysmal nerve pain':ab,ti | 0 |
| #44 | 'pains, nerve':ab,ti | 2 |
| #43 | 'pain, nerve':ab,ti | 235 |
| #42 | 'nerve pains':ab,ti | 8 |
| #41 | 'nerve pain':ab,ti | 624 |
| #40 | 'pains, neuropathic':ab,ti | 2 |
| #39 | 'pain, neuropathic':ab,ti | 556 |
| #38 | 'neuropathic pains':ab,ti | 262 |
| #37 | 'neuropathic pain':ab,ti | 39997 |
| #36 | 'neurodynias':ab,ti | 0 |
| #35 | 'neurodynia':ab,ti | 3 |
| #34 | 'neuralgias':ab,ti | 899 |
| #33 | 'neuralgia':ab,ti | 20684 |
| #32 | 'neuralgia'/exp | 144156 |
| #31 | #1 OR #2 OR #3 OR #4 OR #5 OR #6 OR #7 OR #8 OR #9 OR #10 OR #11 OR #12 OR #13 OR #14 OR #15 OR #16 OR #17 OR #18 OR #19 OR #20 OR #21 OR #22 OR #23 OR #24 OR #25 OR #26 OR #27 OR #28 OR #29 OR #30 | 436243 |
| #30 | 'diabetes mellitus, noninsulin-dependent':ab,ti | 5 |
| #29 | 'diabetes, type 2':ab,ti | 3031 |
| #28 | 'type 2 diabetes':ab,ti | 274535 |
| #27 | 'maturity onset diabetes':ab,ti | 3267 |
| #26 | 'diabetes, maturity-onset':ab,ti | 63 |
| #25 | 'maturity-onset diabetes':ab,ti | 3267 |
| #24 | 'noninsulin dependent diabetes mellitus':ab,ti | 1070 |
| #23 | 'noninsulin-dependent diabetes mellitus':ab,ti | 1070 |
| #22 | 'type 2 diabetes mellitus':ab,ti | 96960 |
| #21 | 'slow-onset diabetes mellitus':ab,ti | 0 |
| #20 | 'diabetes mellitus, slow onset':ab,ti | 1 |
| #19 | 'diabetes mellitus, slow-onset':ab,ti | 1 |
| #18 | 'maturity onset diabetes mellitus':ab,ti | 174 |
| #17 | 'maturity-onset diabetes mellitus':ab,ti | 174 |
| #16 | 'diabetes mellitus, maturity-onset':ab,ti | 15 |
| #15 | 'diabetes mellitus, noninsulin dependent':ab,ti | 5 |
| #14 | 'diabetes mellitus, type ii':ab,ti | 1473 |
| #13 | 'stable diabetes mellitus':ab,ti | 27 |
| #12 | 'diabetes mellitus, stable':ab,ti | 17 |
| #11 | 'non-insulin-dependent diabetes mellitus':ab,ti | 7924 |
| #10 | 'diabetes mellitus, non-insulin-dependent':ab,ti | 43 |
| #9 | 'diabetes mellitus, non insulin dependent':ab,ti | 43 |
| #8 | 'ketosis-resistant diabetes mellitus':ab,ti | 2 |
| #7 | 'diabetes mellitus, ketosis resistant':ab,ti | 0 |
| #6 | 'diabetes mellitus, ketosis-resistant':ab,ti | 0 |
| #5 | 'diabetes mellitus, adult onset':ab,ti | 3 |
| #4 | 'adult-onset diabetes mellitus':ab,ti | 194 |
| #3 | 'diabetes mellitus, adult-onset':ab,ti | 3 |
| #2 | 'non insulin dependent diabetes mellitus':ab,ti | 7922 |
| #1 | 'non insulin dependent diabetes mellitus'/exp | 388054 |

**Table S3 ：Cochrane library search strategy**

ID Search Hits

#1 MeSH descriptor: [Diabetes Mellitus, Type 2] explode all trees 26834

#2 (Diabetes Mellitus, Type 2):ti,ab,kw OR (Diabetes Mellitus, Adult-Onset):ti,ab,kw OR (Adult-Onset Diabetes Mellitus):ti,ab,kw OR (Diabetes Mellitus, Adult Onset):ti,ab,kw OR (Diabetes Mellitus, Ketosis-Resistant):ti,ab,kw 56806

#3 (Diabetes Mellitus, Ketosis Resistant):ti,ab,kw OR (Ketosis-Resistant Diabetes Mellitus):ti,ab,kw OR (Diabetes Mellitus, Non Insulin Dependent):ti,ab,kw OR (Diabetes Mellitus, Non-Insulin-Dependent):ti,ab,kw OR (Non-Insulin-Dependent Diabetes Mellitus):ti,ab,kw 24287

#4 (Diabetes Mellitus, Stable):ti,ab,kw OR (Stable Diabetes Mellitus):ti,ab,kw OR (Diabetes Mellitus, Type II):ti,ab,kw OR (Diabetes Mellitus, Noninsulin Dependent):ti,ab,kw OR (Diabetes Mellitus, Maturity-Onset):ti,ab,kw 10089

#5 (Maturity-Onset Diabetes Mellitus):ti,ab,kw OR (Maturity Onset Diabetes Mellitus):ti,ab,kw OR (Diabetes Mellitus, Slow-Onset):ti,ab,kw OR (Diabetes Mellitus, Slow Onset):ti,ab,kw OR (Slow-Onset Diabetes Mellitus):ti,ab,kw 153

#6 (Type 2 Diabetes Mellitus):ti,ab,kw OR (Noninsulin-Dependent Diabetes Mellitus):ti,ab,kw OR (Noninsulin Dependent Diabetes Mellitus):ti,ab,kw OR (Maturity-Onset Diabetes):ti,ab,kw OR (Diabetes, Maturity-Onset):ti,ab,kw 56297

#7 (Maturity Onset Diabetes):ti,ab,kw OR (Type 2 Diabetes):ti,ab,kw OR (Diabetes, Type 2):ti,ab,kw OR (Diabetes Mellitus, Noninsulin-Dependent):ti,ab,kw 64316

#8 #1 or #2 or #3 or #4 or #5 or #6 or #7 68441

#9 MeSH descriptor: [Neuralgia] explode all trees 2502

#10 (Neuralgia):ti,ab,kw OR (Neuralgias):ti,ab,kw OR (Neurodynia):ti,ab,kw OR (Neurodynias):ti,ab,kw OR (Neuropathic Pain):ti,ab,kw 7662

#11 (Neuropathic Pains):ti,ab,kw OR (Pain, Neuropathic):ti,ab,kw OR (Pains, Neuropathic):ti,ab,kw OR (Nerve Pain):ti,ab,kw OR (Nerve Pains):ti,ab,kw 26463

#12 (Pain, Nerve):ti,ab,kw OR (Pains, Nerve):ti,ab,kw OR (Paroxysmal Nerve Pain):ti,ab,kw OR (Nerve Pain, Paroxysmal):ti,ab,kw OR (Nerve Pains, Paroxysmal):ti,ab,kw 22367

#13 (Pain, Paroxysmal Nerve):ti,ab,kw OR (Pains, Paroxysmal Nerve):ti,ab,kw OR (Paroxysmal Nerve Pains):ti,ab,kw OR (Neuralgia, Atypical):ti,ab,kw OR (Atypical Neuralgia):ti,ab,kw 59

#14 (Atypical Neuralgias):ti,ab,kw OR (Neuralgias, Atypical):ti,ab,kw OR (Neuralgia, Iliohypogastric Nerve):ti,ab,kw OR (Iliohypogastric Nerve Neuralgia):ti,ab,kw OR (Iliohypogastric Nerve Neuralgias):ti,ab,kw 6

#15 (Nerve Neuralgia, Iliohypogastric):ti,ab,kw OR (Nerve Neuralgias, Iliohypogastric):ti,ab,kw OR (Neuralgias, Iliohypogastric Nerve):ti,ab,kw OR (Neuralgia, Ilioinguinal):ti,ab,kw OR (Ilioinguinal Neuralgia):ti,ab,kw 16

#16 (Ilioinguinal Neuralgias):ti,ab,kw OR (Neuralgias, Ilioinguinal):ti,ab,kw OR (Neuralgia, Perineal):ti,ab,kw OR (Neuralgias, Perineal):ti,ab,kw OR (Perineal Neuralgia):ti,ab,kw 16

#17 (Perineal Neuralgias):ti,ab,kw OR (Neuralgia, Stump):ti,ab,kw OR (Neuralgias, Stump):ti,ab,kw OR (Stump Neuralgia):ti,ab,kw OR (Stump Neuralgias):ti,ab,kw 8

#18 (Neuralgia, Supraorbital):ti,ab,kw OR (Neuralgias, Supraorbital):ti,ab,kw OR (Supraorbital Neuralgia):ti,ab,kw OR (Supraorbital Neuralgias):ti,ab,kw OR (Neuralgia, Vidian):ti,ab,kw 18

#19 (Neuralgias, Vidian):ti,ab,kw OR (Vidian Neuralgia):ti,ab,kw OR (Vidian Neuralgias):ti,ab,kw 1

#20 #9 or #10 or #11 or #12 or #13 or #14 or #15 or #16 or #17 or #18 or #19 28737

#21 MeSH descriptor: [Risk Factors] explode all trees 38551

#22 (Risk Factors):ti,ab,kw OR (Factor, Risk):ti,ab,kw OR (Risk Factor):ti,ab,kw OR (Population at Risk):ti,ab,kw OR (Populations at Risk):ti,ab,kw 160689

#23 (Risk Scores):ti,ab,kw OR (Risk Score):ti,ab,kw OR (Score, Risk):ti,ab,kw OR (Risk Factor Scores):ti,ab,kw OR (Risk Factor Score):ti,ab,kw 56137

#24 (Score, Risk Factor):ti,ab,kw OR (Health Correlates):ti,ab,kw OR (Correlates, Health):ti,ab,kw OR (Social Risk Factors):ti,ab,kw OR (Factor, Social Risk):ti,ab,kw 16500

#25 (Factors, Social Risk):ti,ab,kw OR (Risk Factor, Social):ti,ab,kw OR (Risk Factors, Social):ti,ab,kw OR (Social Risk Factor):ti,ab,kw 7434

#26 #21 or #22 or #23 or #24 or #25 189431

#27 #8 and #20 and #26 59

**Table S4 ：Web of science search strategy**

1 TS=(Diabetes Mellitus, Type 2) OR TS=(Diabetes Mellitus, Adult-Onset) OR TS=(Adult-Onset Diabetes Mellitus) OR TS=(Diabetes Mellitus, Adult Onset) OR TS=(Diabetes Mellitus, Ketosis-Resistant) OR TS=(Diabetes Mellitus, Ketosis Resistant) OR TS=(Ketosis-Resistant Diabetes Mellitus) OR TS=(Diabetes Mellitus, Non Insulin Dependent) OR TS=(Diabetes Mellitus, Non-Insulin-Dependent) OR TS=(Non-Insulin-Dependent Diabetes Mellitus) OR TS=(Diabetes Mellitus, Stable) OR TS=(Stable Diabetes Mellitus) OR TS=(Diabetes Mellitus, Type II) OR TS=(Diabetes Mellitus, Noninsulin Dependent) OR TS=(Diabetes Mellitus, Maturity-Onset) OR TS=(Maturity-Onset Diabetes Mellitus) OR TS=(Maturity Onset Diabetes Mellitus) OR TS=(Diabetes Mellitus, Slow-Onset) OR TS=(Diabetes Mellitus, Slow Onset) OR TS=(Slow-Onset Diabetes Mellitus) OR TS=(Type 2 Diabetes Mellitus) OR TS=(Noninsulin-Dependent Diabetes Mellitus) OR TS=(Noninsulin Dependent Diabetes Mellitus) OR TS=(Maturity-Onset Diabetes) OR TS=(Diabetes, Maturity-Onset) OR TS=(Maturity Onset Diabetes) OR TS=(Type 2 Diabetes) OR TS=(Diabetes, Type 2) OR TS=(Diabetes Mellitus, Noninsulin-Dependent) 311,108

2 TS=(Neuralgia) OR TS=(Neuralgias) OR TS=(Neurodynia) OR TS=(Neurodynias) OR TS=(Neuropathic Pain) OR TS=(Neuropathic Pains) OR TS=(Pain, Neuropathic) OR TS=(Pains, Neuropathic) OR TS=(Nerve Pain) OR TS=(Nerve Pains) OR TS=(Pain, Nerve) OR TS=(Pains, Nerve) OR TS=(Paroxysmal Nerve Pain) OR TS=(Nerve Pain, Paroxysmal) OR TS=(Nerve Pains, Paroxysmal) OR TS=(Pain, Paroxysmal Nerve) OR TS=(Pains, Paroxysmal Nerve) OR TS=(Paroxysmal Nerve Pains) OR TS=(Neuralgia, Atypical) OR TS=(Atypical Neuralgia) OR TS=(Atypical Neuralgias) OR TS=(Neuralgias, Atypical) OR TS=(Neuralgia, Iliohypogastric Nerve) OR TS=(Iliohypogastric Nerve Neuralgia) OR TS=(Iliohypogastric Nerve Neuralgias) OR TS=(Nerve Neuralgia, Iliohypogastric) OR TS=(Nerve Neuralgias, Iliohypogastric) OR TS=(Neuralgias, Iliohypogastric Nerve) OR TS=(Neuralgia, Ilioinguinal) OR TS=(Ilioinguinal Neuralgia) OR TS=(Ilioinguinal Neuralgias) OR TS=(Neuralgias, Ilioinguinal) OR TS=(Neuralgia, Perineal) OR TS=(Neuralgias, Perineal) OR TS=(Perineal Neuralgia) OR TS=(Perineal Neuralgias) OR TS=(Neuralgia, Stump) OR TS=(Neuralgias, Stump) OR TS=(Stump Neuralgia) OR TS=(Stump Neuralgias) OR TS=(Neuralgia, Supraorbital) OR TS=(Neuralgias, Supraorbital) OR TS=(Supraorbital Neuralgia) OR TS=(Supraorbital Neuralgias) OR TS=(Neuralgia, Vidian) OR TS=(Neuralgias, Vidian) OR TS=(Vidian Neuralgia) OR TS=(Vidian Neuralgias) 114,505

3 TS=(Risk Factors) OR TS=(Factor, Risk) OR TS=(Risk Factor) OR TS=(Population at Risk) OR TS=(Populations at Risk) OR TS=(Risk Scores) OR TS=(Risk Score) OR TS=(Score, Risk) OR TS=(Risk Factor Scores) OR TS=(Risk Factor Score) OR TS=(Score, Risk Factor) OR TS=(Health Correlates) OR TS=(Correlates, Health) OR TS=(Social Risk Factors) OR TS=(Factor, Social Risk) OR TS=(Factors, Social Risk) OR TS=(Risk Factor, Social) OR TS=(Risk Factors, Social) OR TS=(Social Risk Factor) 233,8896

4 #1 AND #2 AND #3 193

**Table S5 Single factor meta-analysis**

| Risk factors | The studies mentioned | heterogeneity | | OR/WMD (95%CI) | P | Egger |
| --- | --- | --- | --- | --- | --- | --- |
|  |  | I^2^(%) | P |  |  |  |
| Female gender | 14 | 72.3 | 0.000 | 1.29(1.09, 1.54) | 0.004 | 0.022 |
| Age of onset | 13 | 87.2 | 0.000 | 1.25(-0.21, 2.70) | 0.093 | 0.018 |
| BMI | 8 | 76.3 | 0.000 | 0.56 (-0.02, 1.14) | 0.059 | 0.102 |
| Glycosylated hemoglobin levels | 11 | 26.1 | 0.196 | 0.14 (0.09, 0.19) | 0.000 | 0.188 |
| Duration of diabetes | 11 | 89.9 | 0.000 | 1.28 (-0.24, 2.79) | 0.099 | 0.439 |
| Concomitant nephropathy | 7 | 31.0 | 0.191 | 1.41 (1.34, 1.49) | 0.000 | 0.011 |
| Associated retinopathy | 7 | 65.5 | 0.008 | 1.32 (1.01, 1.71) | 0.040 | 0.511 |
| Concomitant cardiovascular disease | 8 | 57.8 | 0.020 | 1.46 (1.19, 1.80) | 0.000 | 0.005 |
| Arterial hypertension | 12 | 73.1 | 0.000 | 1.25 (1.00, 1.55) | 0.047 | 0.377 |
| Dyslipidemia levels | 5 | 52.1 | 0.079 | 1.41(0.92, 2.17) | 0.118 | 0.970 |
| Triglycerides levels | 5 | 50.0 | 0.091 | -0.10 (-0.13,-0.08 ) | 0.000 | 0.585 |
| Cholesterol levels | 5 | 68.4 | 0.013 | -0.09 (-0.21,0.04) | 0.170 | 0.133 |
| High density lipoprotein levels | 5 | 0.0 | 0.935 | 0.00（-0.02，0.02） | 0.962 | 0.964 |
| Low density lipoprotein levels | 4 | 27.6 | 0.251 | -0.20（-0.22，-0.17） | 0.000 | 0.230 |
| Smoking | 8 | 0.0 | 0.564 | 0.90（0.84，0.95） | 0.001 | 0.924 |
| Drinking alcohol | 3 | 27.6 | 0.251 | 0.87（0.80，0.95） | 0.001 | 0.306 |
| Glomerular filtration rate levels | 5 | 23.2 | 0.267 | -7.11（-8.03，-6.20） | 0.000 | 0.822 |
| Retirement | 6 | 80.0 | 0.000 | 1.15（0.72，1.81） | 0.559 | 0.244 |
| Work regularly | 6 | 69.3 | 0.006 | 0.80（0.49，1.31） | 0.374 | 0.010 |
| Oral hypoglycemic agents | 6 | 79.3 | 0.000 | 0.84（0.64，1.09） | 0.186 | 0.606 |
| Lipid-lowering Agents | 3 | 0.0 | 0.711 | 0.96(0.76,1.20) | 0.714 | 0.806 |
| Insulin Therapy | 8 | 63.4 | 0.008 | 1.00(0.80,1.25) | 0.984 | 0.358 |
| Diabetic Foot ulcers | 3 | 0.0 | 0.404 | 1.34(0.85,2.12) | 0.208 | 0.404 |
| Obesity | 5 | 60.4 | 0.056 | 2.00(1.33,3.02) | 0.001 | 0.286 |
| Sports and Exercise | 3 | 82.4 | 0.003 | 0.74(0.50,1.08) | 0.118 | 0.463 |
| Serum creatinine levels | 3 | 0.0 | 0.442 | 0.40(-0.20,1.00) | 0.193 | 0.559 |

**Table S6 Multi-factor meta-analysis**

| Risk factors | The studies mentioned | heterogeneity | | OR (95%CI) | P | Egger |
| --- | --- | --- | --- | --- | --- | --- |
|  |  | I^2^(%) | P |  |  |  |
| Female gender | 5 | 82.4 | 0.000 | 1.42 (1.03, 1.97) | 0.032 | 0.969 |
| Age of onset | 7 | 95.2 | 0.000 | 1.19 (1.11, 1.27) | 0.000 | 0.008 |
| Duration of diabetes | 6 | 98.0 | 0.000 | 1.29 (1.15,1.45 ) | 0.000 | 0.076 |
| Concomitant nephropathy | 3 | 97.9 | 0.000 | 1.62 (0.89, 2.96) | 0.114 | 0.728 |
| Associated retinopathy | 3 | 98.2 | 0.000 | 1.90 (1.06,3.40 ) | 0.032 | 0.401 |
| Arterial hypertension | 5 | 97.3 | 0.000 | 1.12 (0.75, 1.70) | 0.575 | 0.521 |
| Smoking | 3 | 84.1 | 0.002 | 1.24(0.57, 2.68) | 0.583 | 0.633 |

**Table S7 Diagnostic criteria for painful diabetic neuropathy**

| study | country | Diagnostic criteria for painful diabetic neuropathy | Regression model |
| --- | --- | --- | --- |
| David Kec2022 | Czech Republic | According to the predefined cut-off for pre-treatment pain intensity(NRS≥4) and other criteria for chronic neuropathic pain | multiple regression |
| Pai Yen-wei2017 | Taiwan of China | The diagnosis of neuropathic pain was made, using validated neuropathic pain screening tool DN4 questionnaire, if the score is more than or equal to 4 | logistic regression |
| Ponirakis2019 | Qatar | A score≥4has a high sensitivity (80%) and speciﬁcity (92%) for  PDPN | logistic regression |
| Chenxi2023 | mainland China | The diagnosis of PDPN was made if the pain was bilateral, below the  knees, often worse at night, unrelated to exertion, and not caused by BMI、SBP、TG and so on. | logistic regression |
| Alamri2021 | Riyadh,Saudi Arabia | The Neuropathy Total Symptom Score-6  (NTSS-6) was used to estimate the frequency of neuropathic symptoms | logistic regression |
| Weng2020 | Taiwan of China | DPNP in this study was deﬁned as a total DN4-T score of 4 or more out of 10 in both lower legs, excluding those with neuralgia noted  only in the hand or related carpal tunnel syndrome. | logistic regression |
| Celik2015 | Turkish | Those patients who had a DN4 score≥4 were considered to have PDN | logistic regression |
| Ying2017 | Malaysian | The pain DETECT questionnaire(PD-Q) was  used to screen for the presence of neuropathic pain symptoms, with a score of >19 indicating that a neuropathic pain component is likely | logistic regression |
| Elliott2024 | Europe | Painful DPN was deemed present if a participant had evidence of distal symmetrical polyneuropathy (according to the protocol) with painful neu-ropathic symptoms (deep aching or burning pains) in the distribution of the peripheral neuropathy | logistic regression |
| jacovides2014 | South Africa | DPNP was diagnosed using Douleur Neuropathique 4 (DN4) questionnaire (score≥4) | logistic regression |
| Liau2022 | Taiwan of China | DPNP was diagnosed using Douleur Neuropathique 4 (DN4) questionnaire (score≥4) | Cox regression |
| naranjo2020 | Spain | DPNP was diagnosed using Douleur Neuropathique 4 (DN4) questionnaire (score≥4) | logistic regression |
| Palomo2022 | Spain | The neuropathic pain phenotype was assessed by means of the Neuropathic Pain Symptoms Inventory (NPSI). | logistic regression |
| Raputova2017 | Czech Republic | The Neuropathic Pain Symptom Inventory  (NPSI), a self-administered questionnaire, was applied to evaluate NeuP symptoms. We used the Czech and German validated versions of the NPSI | logistic regression |

**Table S8 Table of literature characteristics**

| study | country | study design | sample size | | Gender(M/F) | PDN/ non-PDN | | | Age(years) | |  |  |
| --- | --- | --- | --- | --- | --- | --- | --- | --- | --- | --- | --- | --- |
|  |  |  | S | NS |  | M/F | | M/F | S | NS |  |  |
| David Kec2022 | Czech Republic | a cohort study | 347 | 311 | 374/284 | 194/153 | | 176/135 | 59.2±10.7 | 57.9±12.1 |  |  |
| Pai Yen-wei2017 | Taiwan of China | a cross-sectional study | 128 | 476 | 361/243 | 63/65 | | 296/178 | 71.6±12.4 | 71.2±12.2 |  |  |
| Ponirakis2019 | Qatar | a cohort study | 378 | 717 | 659/430 | 206/169 | | 453/261 | 57.5±10.7 | 52.6±11.4 |  |  |
| Chenxi2023 | mainland China | a cross-sectional study | 14699 | 11011 | 14925/10785 | 8459/6240 | 6466/4545 | | 64.7±12.6 | 61±11.9 |  |  |
| Alamri2021 | Riyadh,Saudi Arabia | a cross-sectional study | 182 | 103 | 129/156 | 79/103 | | 50/53 | 0-96 | |  |  |
| Weng2020 | Taiwan of China | a cross-sectional study | 16 | 129 | 91/54 | 10/6 | | 81/48 | 54.6±11.1 | 57.5±8.1 |  |  |
| Celik2015 | Turkish | a cross-sectional study | 313 | 1044 | 590/767 | 159/154 | | 431/613 | 59.32±11.48 | 57.86±12.25 |  |  |
| Ying2017 | Malaysian | a cohort study | 13 | 229 | 102/140 | 5/8 | | 97/132 | 56.2±12.2 | 61.2±11.3 |  |  |
| Elliott2024 | Europe | a cohort study | 41 | 235 | 124/152 | 11/30 | | 113/122 | 35±10.1 | 33.4±9.9 |  |  |
| jacovides2014 | South Africa | a cross-sectional study | 291 | 670 | 535/510 | 126/165 | | 365/304 | 57.3±11.8 | 55.3±13.7 |  |  |
| Liau2022 | Taiwan of China | a cohort study | 179 | 2139 | 1307/1011 | 82/97 | 1225/914 | | 68.7±13.2 | 62.6±13.1 |  |  |
| naranjo2020 | Spain | a cross-sectional study | 65 | 65 | 63/67 | 27/38 | 36/29 | | 70.25±10.02 | 74.75±8.97 |  |  |
| Palomo2022 | Spain | a cross-sectional study | 71 | 78 | 83/66 | 36/35 | 47/31 | | 69.55±9.74 | 73.35±8.2 |  |  |
| Raputova2017 | Czech Republic | a cross-sectional study | 106 | 74 | 107/73 | 54/52 | 33/19 | | 60.2±29.6 | 57.3±38.7 |  |  |

S: Diabetic peripheral with pain; NS: Diabetic peripheral without pain

**Table S9 NOS scores**

| Study | Is the case definition adequate? | Representativeness of the cases | Definition of Controls | Comparability of cases and controls based on the design or analysis | Ascertainment of exposure | Same method of ascertainment for cases and controls | Non response | Total scores |
| --- | --- | --- | --- | --- | --- | --- | --- | --- |
| David Kec2022 | * | * | * | * | * | * | * | 7 |
| Pai Yen-wei2017 | * | * | * | * | * | * | - | 6 |
| Ponirakis2019 | * | * | * | * | * | * | * | 7 |
| Chenxi2023 | * | * | * | * | * | * | * | 7 |
| Alamri2021 | * | * | * | * | * | * | * | 7 |
| Weng2020 | * | * | * | * | * | * | * | 7 |
| Celik2015 | * | * | * | * | * | * | * | 7 |
| Ying2017 | * | * | * | * | * | * | * | 7 |
| Elliott2024 | * | * | * | ** | * | * | * | 8 |
| jacovides2014 | * | * | * | ** | * | * | * | 8 |
| Liau2022 | * | * | * | - | * | * | * | 6 |
| naranjo2020 | * | * | * | ** | * | * | * | 8 |
| Palomo2022 | * | * | * | * | * | * | * | 7 |
| Raputova2017 | * | * | * | * | * | * | * | 7 |
