# Supplementary material for: Prevalence and risk factors for painful diabetic peripheral neuropathy: a systematic review and meta-analysis
Source: Front Neurol. 2025 May 13;16:1564867. doi: 10.3389/fneur.2025.1564867 (PMC12108811; doi:10.3389/fneur.2025.1564867)
Supplement: Supplementary file 2 [file Supplementary_file_2.docx]

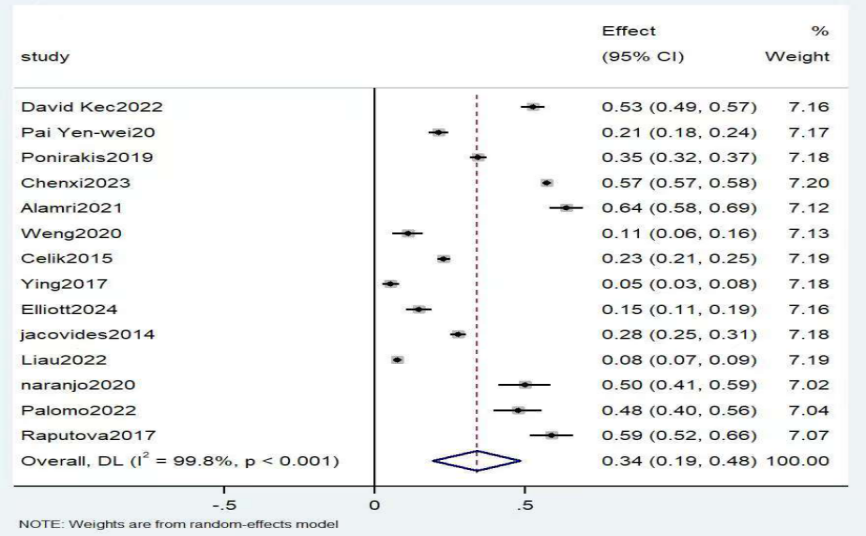


Figure 1 : Forest plot of prevalence subgroup analysis


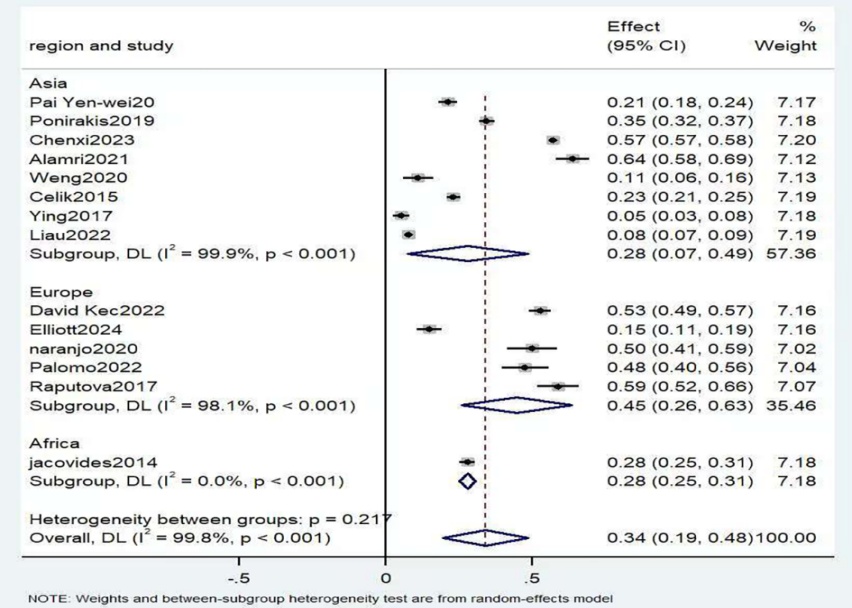


Figure 2 : Forest plot of region subgroup analysis


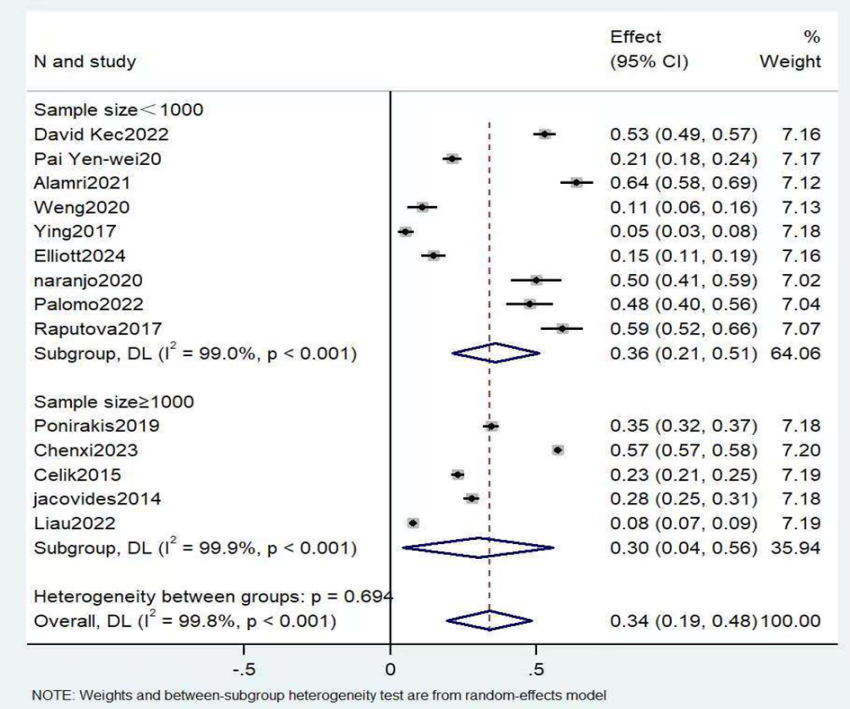


Figure 3 : Forest plot of sample size subgroup analysis


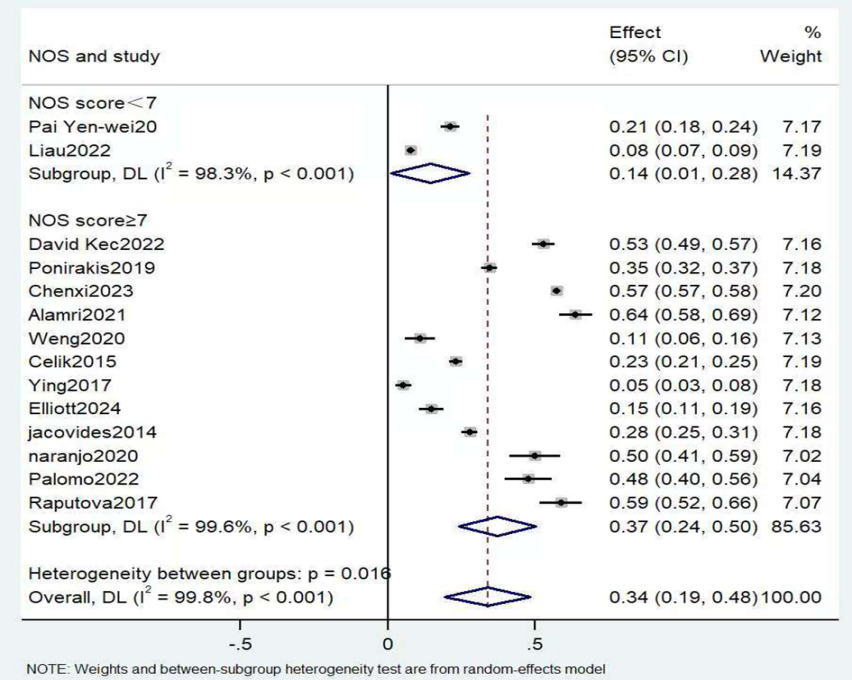


Figure 4 : Forest plot of NOS subgroup analysis


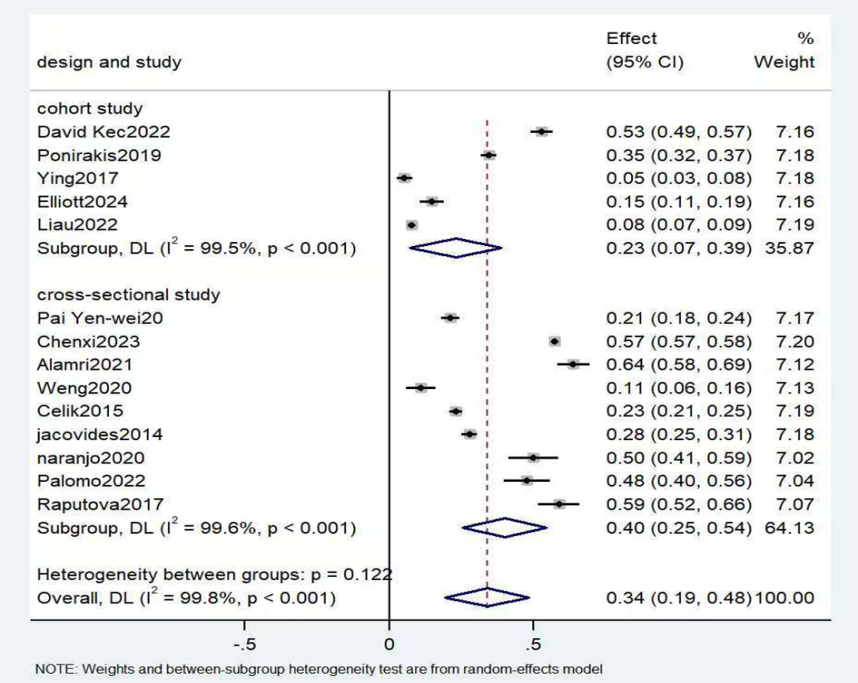


Figure 5 : Forest plot of design subgroup analysis


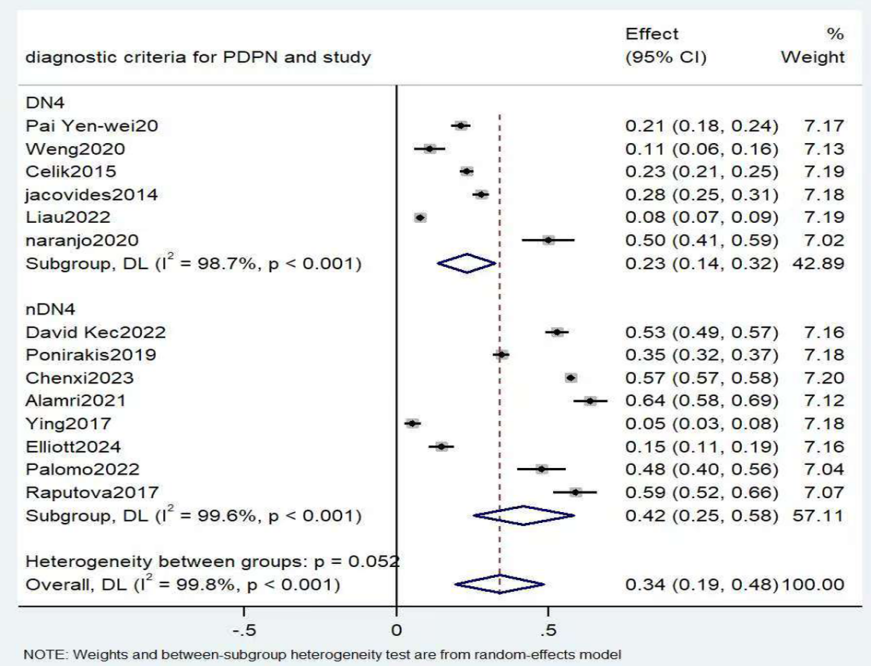


Figure 6 : Forest plot of PDPN subgroup analysis


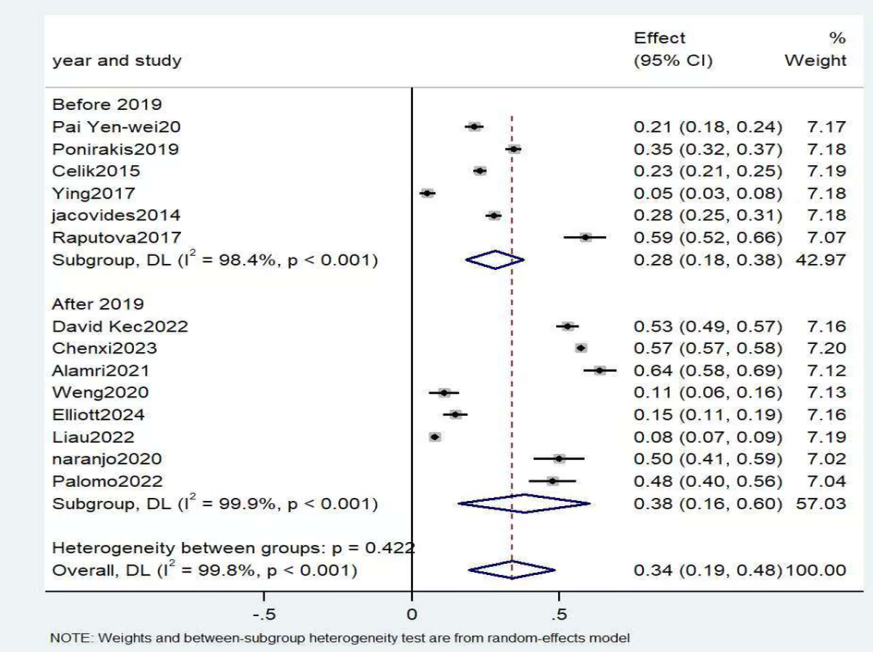


Figure 7 : Forest plot of year subgroup analysis


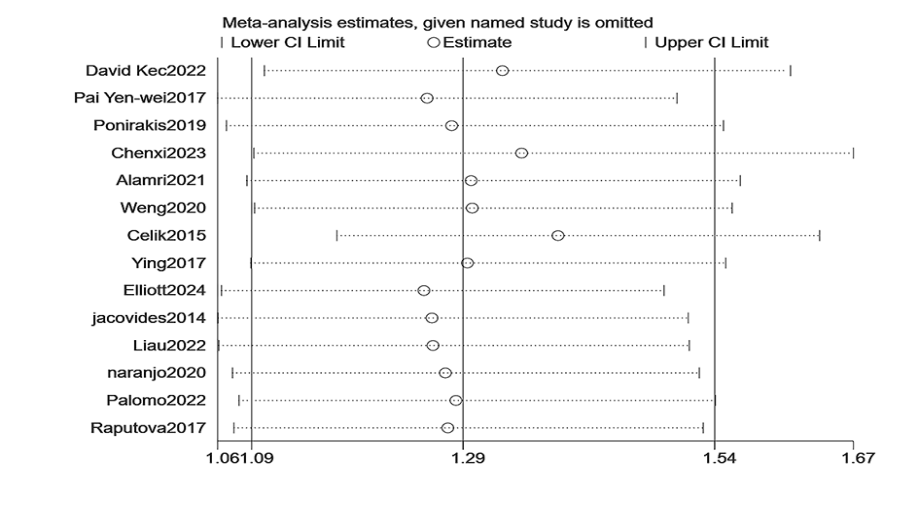


Figure 8 : Sensitivity sensitivity analysis in Women


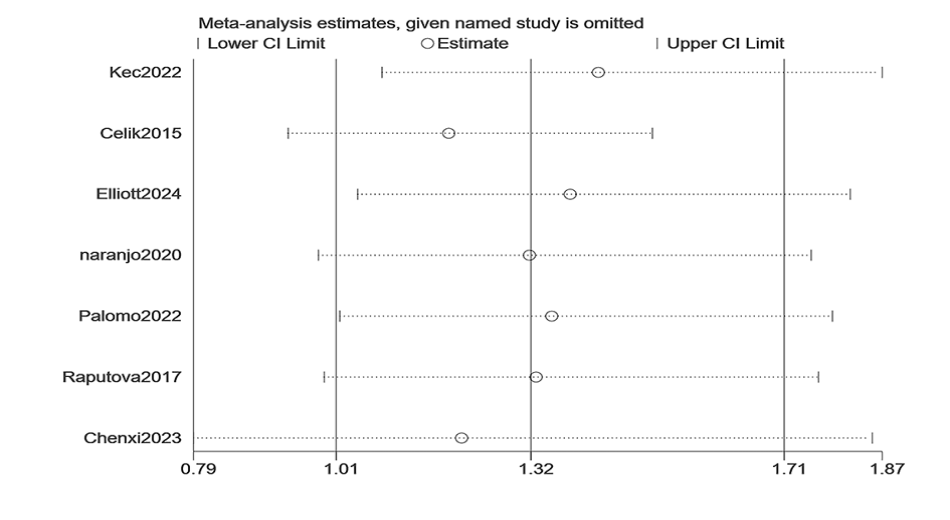


Figure 9 : Sensitivity sensitivity analysis in Associated retinopathy


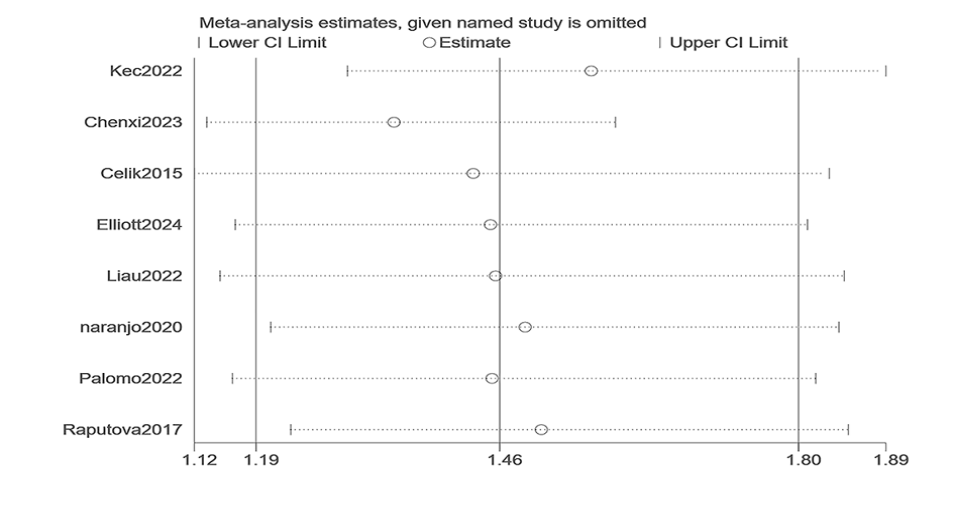


Figure 10 : Sensitivity sensitivity analysis in Concomitant cardiovascular disease


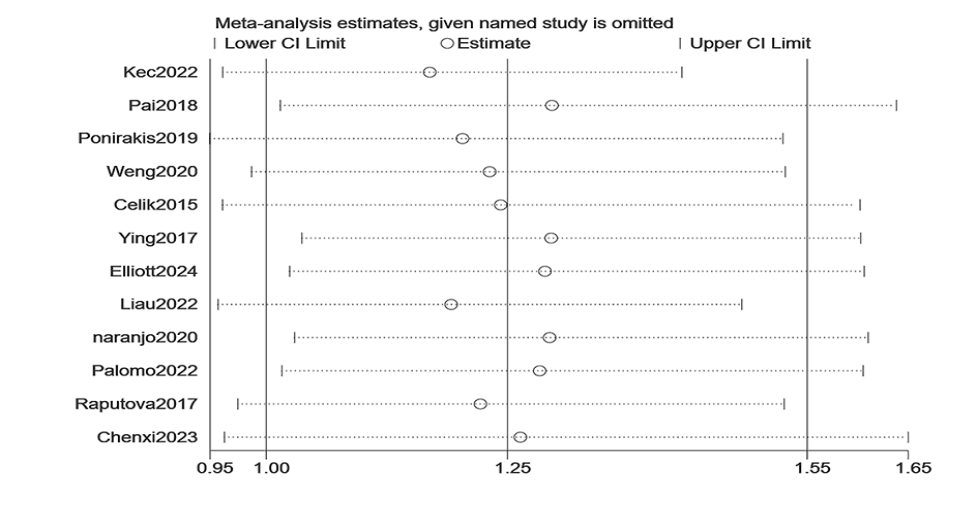


Figure 11 : Sensitivity sensitivity analysis in Arterial hypertension


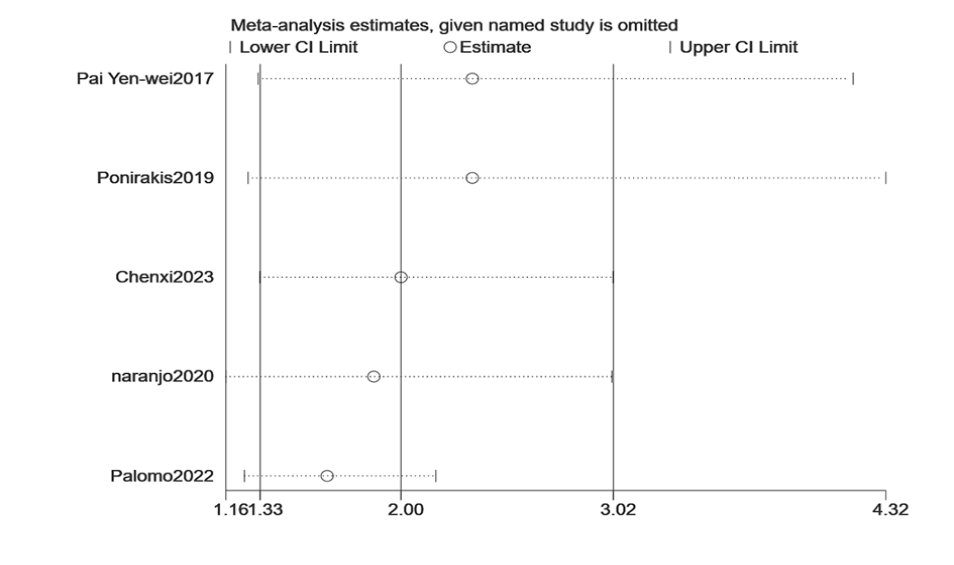


Figure 12 : Sensitivity sensitivity analysis in Obesity
